# Protein characteristics substantially influence the propensity of activity cliffs among kinase inhibitors

**DOI:** 10.1038/s41598-024-59501-w

**Published:** 2024-04-20

**Authors:** Safa Daoud, Mutasem Taha

**Affiliations:** 1 Department of Pharmaceutical Chemistry and Pharmacognosy, Faculty of Pharmacy, Applied Sciences Private University, Amman, Jordan; 2 Department of Pharmaceutical Sciences, Faculty of Pharmacy, University of Jordan, Amman, Jordan

**Keywords:** Computational biology and bioinformatics, Drug discovery

## Abstract

Activity cliffs (ACs) are pairs of structurally similar molecules with significantly different affinities for a biotarget, posing a challenge in computer-assisted drug discovery. This study focuses on protein kinases, significant therapeutic targets, with some exhibiting ACs while others do not despite numerous inhibitors. The hypothesis that the presence of ACs is dependent on the target protein and its complete structural context is explored. Machine learning models were developed to link protein properties to ACs, revealing specific tripeptide sequences and overall protein properties as critical factors in ACs occurrence. The study highlights the importance of considering the entire protein matrix rather than just the binding site in understanding ACs. This research provides valuable insights for drug discovery and design, paving the way for addressing ACs-related challenges in modern computational approaches.

## Introduction

Activity cliffs (ACs) are pairs of closely similar molecules that have significantly dissimilar affinities towards certain biotarget^1^. The prevalence of ACs in SAR data^2^ necessitates that modern computer-assisted drug discovery and design effectively address this issue^3–9^. Moreover, ACs pose substantial challenge for bioactivity-supervised discovery approaches that rely on smooth and continuous structure–activity correlations^10^.

A number of machine learning computational approaches have been evaluated to forecast ACs pairs using ligand patterns^2,11–13^ or target-based pharmacophores^14^. ACs are conventionally described to be caused by subtle local differences in the 3D enthalpic contacts of cliff-forming ligands within the binding site^15^. In this direction, molecular dynamics and free energy perturbation were utilized to explain ACs^16–20^, nonetheless with affinity prediction errors^21^ (Tables 1, 2).

**Table 1:**
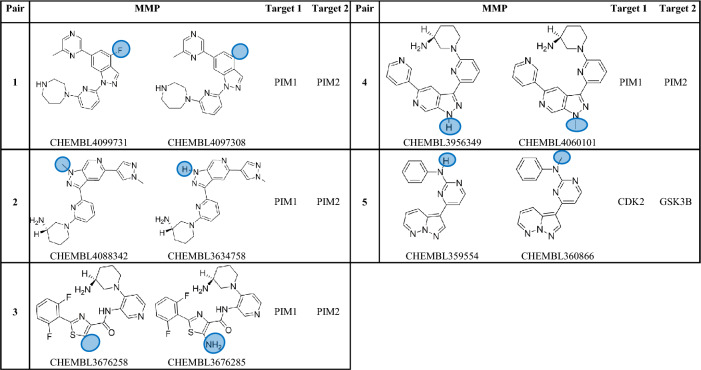
Matched Molecular Pairs (MMPs) exclusively manifested as ACs among kinase populations that are included in this study.

**Table 2:** Collected kinases, their UniProt code, count of amino Acid (AA), corresponding used crystal structures, collected inhibitors, MMPs, Acs and % ACs/MMPs.

Protein kinase	UniProt code	AA count	Crystal structure count	Inhibitors count	MMPs count	ACs count	ACs/MMPs%	Protein kinase	UniProt code	AA count	Crystal structure count	Inhibitors count	MMPs count	ACs count	ACs/MMPs%
ABL1*	P00519	1130	12	1414	858	24	2.8	ITK*	Q08881	620	8	736	181	1	0.6
ACK1(TNK2)	Q07912	1038	7	376	54	1	1.9	JAK2	O60674	1132	20	1743	816	19	2.3
AKT2	P31751	481	4	784	201	0	0.0	KDR (VEGFR2)	P35968	1356	14	1276	372	8	2.2
ALK	Q9UM73	1620	18	827	194	0	0.0	KIT(SCFR)	P10721	976	5	688	184	15	8.2
AURKA (Aurora A)*	O14965	403	19	1137	341	5	1.5	KRS1(MST2)	Q13188	491	1	810	228	1	0.4
AXL(UFO)	P30530	894	1	434	87	0	0.0	LCK	P06239	509	20	1761	353	1	0.3
BRK(PTK6)	Q13882	451	2	398	65	1	1.5	LIMK1	P53667	647	4	762	169	0	0.0
BTK	Q06187 Q06187 **Q06187 7 Q06187** Q06187	659	18	764	170	1	0.6	LYN	P07948	512	2	619	171	0	0.0
CAMK2D	Q13557	499	4	626	153	0	0.0	MAP4K4 (HGK)	O95819	1239	14	980	251	8	3.2
CDK2	P24941	298	20	1115	541	21	3.9	MAPK1 (ERK2)*	P28482	360	19	1653	387	2	0.5
CHEK1 (CHK1)	O14757	476	19	1041	272	4	1.5	MAPK13(SAPK4)*	O15264	365	4	730	185	0	0.0
CHEK2	O96017	543	20	675	165	0	0.0	MAPK8 (JNK1)	P45983	427	12	927	220	0	0.0
CLK2	P49760	499	6	886	220	5	2.3	MAPKA(3PK)* (3pK)*	Q16644	382	5	737	192	0	0.0
CLK4	Q9HAZ1	481	1	985	208	4	1.9	MARK2(EMK1)	Q7KZI7	788	3	646	161	0	0.0
CNK(PLK3)	Q9H4B4	646	1	722	184	0	0.0	MELK(PK38)	Q14680	651	18	576	142	5	3.5
CSF1R (FMS)*	P07333	972	7	959	254	11	4.3	MET	P08581	1390	18	1265	352	1	0.3
CSNK1A1 (CK1α1)	P48729	337	1	919	224	0	0.0	NEK2*	P51955	445	19	894	220	0	0.0
CSNK1D	P48730	415	17	733	186	0	0.0	NTRK1 (TRKA)*	P04629	796	17	884	222	1	0.5
CSNK2A1(CK2α1)	P68400	391	19	814	215	6	2.8	NTRK2 (TRKB)	Q16620	822	3	919	234	2	0.9
cTAK1(MARK3)	P27448	753	1	760	202	1	0.5	NTRK3 (TRKC)	Q16288	839	4	680	142	1	0.7
DAPK3 (ZIPK)	O43293	454	5	919	249	2	0.8	PAK1	Q13153	545	8	879	256	0	0.0
DCLK1	O15075	740	5	608	129	0	0.0	PAK4*	O96013	591	13	1197	354	0	0.0
DYRK1A*	Q13627	763	20	1047	275	6	2.2	PDK1	Q15118	436	20	709	179	0	0.0
EGFR (ErbB1)	P00533	1210	17	1557	419	11	2.6	PHKG2(PSK-C3)*	P15735	406	1	580	116	0	0.0
EPHA2(ECK)	P29317	976	17	694	174	1	0.6	PI3K-alpha	P42336	1068	13	1075	672	8	1.2
FAK1(PTK2)	Q05397	1052	19	725	158	0	0.0	PIM1*	P11309	313	17	2498	1372	17	1.2
FES*	P07332	822	1	392	68	0	0.0	PIM2	Q9P1W9	311	2	866	286	5	1.7
FGFR1*	P11362	822	8	986	252	1	0.4	PKN2(PRKCL2)*	Q16513	984	1	701	167	4	2.4
FGFR3(JTK4)	P22607	806	2	657	160	0	0.0	PLK1	P53350	603	5	829	218	1	0.5
FLT1*	P17948	1338	1	1588	286	11	3.8	PRKACA (PKA)*	P17612	351	17	1167	377	0	0.0
FLT3	P36888	993	5	1025	275	7	2.5	PRKCI (PKCiota)	P41743	596	5	479	99	0	0.0
FYN	P06241	537	1	1665	325	2	0.6	PYK2(FAK2)	Q14289	1009	9	427	85	0	0.0
GSK3B (GSK3 beta)	P49841	420	20	1356	529	35	6.6	RET	P07949	1114	17	727	138	3	2.2
HCCS-4	Q9NYL2	800	3	604	125	0	0.0	ROCK1	Q13464	1354	19	1127	382	0	0.0
HER2 (erbB2)	P04626	1255	2	1390	222	0	0.0	RPS6KA3 (RSK2)	P51812	740	8	1000	260	2	0.8
HER4 (ErbB4)*	Q15303	1308	2	670	167	0	0.0	RPS6KB1 (S6K1)	P23443	525	13	716	184	5	2.7
HIPK2	Q9H2X6	1198	2	680	175	0	0.0	SRC	P12931	536	6	1056	547	7	1.3
IGF1R	P08069	1367	11	870	257	0	0.0	SRPK1	Q96SB4	655	6	609	127	0	0.0
INSR	P06213	1382	2	734	183	0	0.0	SYK	P43405	635	18	591	165	0	0.0
IRAK4	Q9NWZ3	460	20	869	264	3	1.1	TYK2	P29597	1187	3	802	283	0	0.0

*These were included in the testing set.

Protein kinases have long been acknowledged as significant therapeutic targets. A number of small molecule kinase inhibitors are in development or have already received approval for the treatment of a number of human ailments, including cancer, cardiovascular problems, and inflammation^22^. However, after careful examining of this group of enzymes, we noticed that while some of these enzymes exhibit several ACs, others appear to be immune to this phenomenon despite having hundreds, or even thousands, of reported inhibitors. Moreover, in many instances closely homologous molecules (matched molecular pairs, MMPs) emerge as ACs upon interacting with some protein kinases while they maintain similar affinities with others, see Table 3 in “Results”.

**Table 3:**
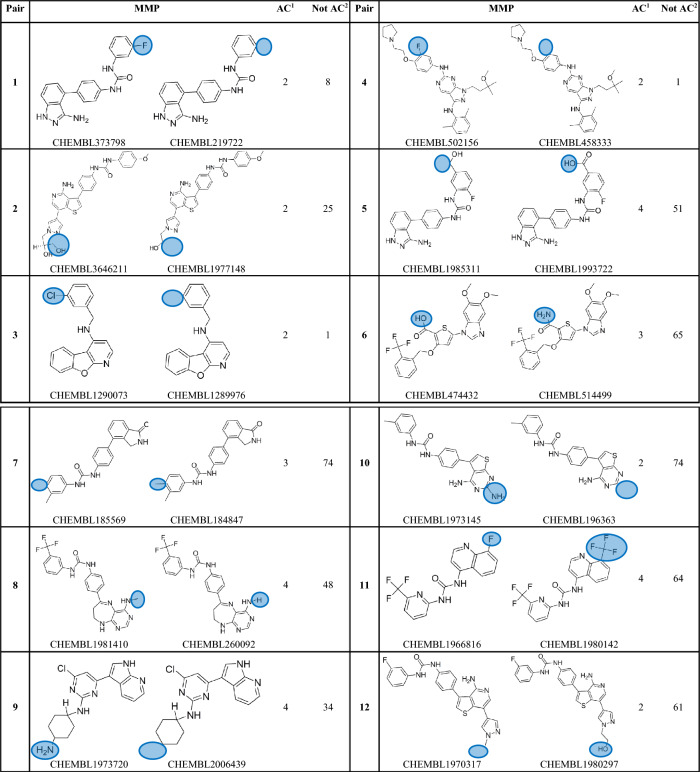

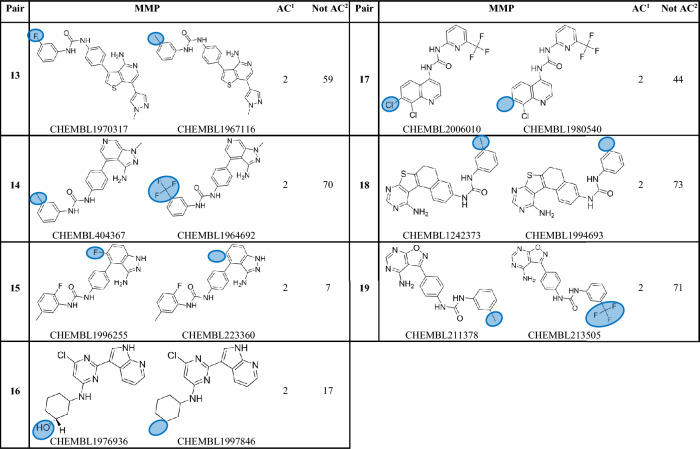
Matched Molecular Pairs (MMPs) frequently found among kinase ligand populations included in this study.

^1^Count of protein kinases in which the particular MMP was reported as activity cliff partners among corresponding ligands.

^2^Count of protein kinases in which the particular MMP was reported to have similar potencies (i.e., not activity cliff partners) among corresponding ligands.

Such observations led us to postulate that the existence of ACs is reliant on the target protein. Moreover, we propose that the propensity to have ACs is related to the complete protein matrix, not just the binding site, because all protein kinases have somehow similar ATP catalytic sites that are often targeted by tailored inhibitors^23^.

This supposition is in line with our recently presented theory, which states that the binding of potent AC members results in large, entropically driven conformational alterations in the target protein, which in turn reveal hidden attractive features within the binding site. These "new" interaction sites offer extra enthalpic binding contributions^24,25^.

To pursue our proposition, we focused on protein kinases of numerous reported inhibitors in ChEMBL database. We then systematically identified ACs within inhibitors population of each protein. We defined ACs as structural analogs with at least 100-fold difference in potency based on well-defined bioactivity measurements^26^. We then generated numerous protein descriptors (*ca.* 9900) for the studied proteins based on their amino acid sequences. Additional descriptors were also extracted from the 3D-crysallographic structures of these proteins. Following that, we ran several machine learning (ML) models to see if we could link protein properties to the presence or absence of ACs in the corresponding ligands population. Moreover, we applied genetic algorithm^27^ to identify the most probable protein descriptors that control the ACs phenomenon.

## Experimental

Machine learning (ML) details, including: training, deployment of different learners, genetic algorithm (GA) selection of descriptors, as well as model assessment using accuracy and Cohen’s kappa values against training and testing sets were performed using KNIME analytics platform (version 4.3.3). All implemented workflows are provided in the Supporting Folder 1. Protein descriptors were calculated using ProtrWeb (http://protr.org/). Three-dimensional protein descriptors were calculated within Discovery Studio (version 4.5, Biovia).

### Collection of protein kinases and generation of protein descriptors

Eighty protein kinases, wild type only, were selected (see Table 2). The amino acid sequences of designated proteins were downloaded from UniProt (https://www.uniprot.org/). Subsequently, numerous structural and physicochemical descriptors were calculated for each protein employing ProtrWeb (http://protr.org/)^28^. ProtrWeb offers twelve groups of protein descriptors including: Amino acid composition, dipeptide composition, tripeptide composition, normalized Moreau-Broto autocorrelation, Moran autocorrelation, Geary autocorrelation, CTD (composition, transition, distribution) descriptors, conjoint triad, sequence-order-coupling number, quasi-sequence-order descriptors, pseudo-amino acid composition and amphiphilic pseudo-amino acid composition^28^. Totally, 9920 descriptors were generated for each protein (see Supporting Excel file SM1).

Furthermore, the Protein Data Bank was searched for crystallographic structures of corresponding kinase domains of collected protein kinases. Only wild type, mutations-free proteins were included. Crystallographic structures corresponding to *apo* proteins were excluded. Moreover, we scrutinized the collected structures against KLIFS database^29^ such that crystallographic complexes involving type II protein kinase inhibitors bound to kinases of DFG-out conformations were also excluded. In case a certain protein is represented by numerous entries in the protein databank, we opted to include a maximum of 20 structures of best possible resolution in the analysis; however, if a particular protein kinase is represented by fewer than 20 entries, all structures were included. Consequently, 771 crystallographic structures were collected (see Supporting Excel file SM1). Co-crystallized ligands and hydration water molecules were removed. Hydrogen atoms were added utilizing the Discovery Studio 4.5 template for hydrogen atoms. The following descriptors were determined for each crystallographic structure using Discovery Studio 4.5: Count of intramolecular hydrogen bonds; count of intramolecular bumps (pair of atoms at close proximity such that they violate each other VDW spheres by at least 30% without being covalently bonded); count of intramolecular electrostatic interactions, and count of hydrophobic interactions. Needless to say that the 3D structure of a protein is controlled by these interactions such that any change in the 3D structures of the protein should be accompanied by the changes in the individual counts of these interactions. Normalized versions of these descriptors were also included. The normalized descriptors were calculated by dividing each descriptor by the number of non-hydrogen atoms in the protein. Scaling 3D descriptors by the number of non-hydrogen atoms in the crystallographic structure addresses a crucial variability in protein sizes used for crystallization. Crystallographers often cleave certain protein fragments to successfully achieve crystallographic structures. Such size variations can significantly influence 3D descriptors, skewing their interpretation. Normalizing 3D descriptors by dividing by the number of heavy atoms (excluding hydrogens) effectively mitigates this size-related bias. Consequently, comparing 3D descriptors across different protein crystallographic structures becomes more meaningful, especially when investigating ligand-induced conformational changes.

For example, if certain protein is represented by 10 crystallographic structures, then ProtrWeb descriptors were calculated once for this protein based on its amino acid sequence. The resulting descriptors were then concatenated ten times with ten different sets of 3D descriptors corresponding to each crystallographic structure of the same protein. Therefore, the section corresponding to this protein in the machine learning training, or testing, tables included 10 row entries filled with the same ProtrWeb descriptors and differing 3D descriptors according to each crystallographic structure. The repetitive use of slightly differing data is commonly implemented in machine learning as data augmentation tool to enhance machine learning models^30–32^. All protein entries and descriptors are shown in supporting Excel file SM1.

### Protein kinase inhibitors

Inhibitors of each collected protein kinase together with their associated bioactivity data were gathered from ChEMBL database (https://www.ebi.ac.uk/chembl/). Only Inhibitors of wild type protein kinases were collected. Being largely bioassay-independent, only Ki values were collected for this study. Molecules of approximate Ki values such as “>”, “<”, or “~” were excluded. For molecules of multiple Ki values, the corresponding geometric average was considered as potency label on the condition that all values fell within the same order of magnitude, else they were discarded.

Protein kinase inhibitors are classified as being type I or type II based on the enzyme activation state during binding bioassay^33,34^. Type I inhibitors typically do not require a DFG-out conformation for binding and they are compatible with multiple A-loop conformations, i.e., type I inhibitors do not exhibit significant activation state preference. In contrast, type II inhibitors bind preferentially to DFG-out conformation^33,34^. Unfortunately, bioactivity data in ChEMBL lack clear and consistent information about the activation state of protein kinases during bioassay conditions. To overcome this obstacle, we included only type I kinase inhibitors in this study. This was possible based on a Hu et al*.* study, which identified 70 molecular fragments as type II signatures^35^. Accordingly, SMARTS depictions of all 70 signature fragments^35^ were generated using Discovery Studio 4.5 and were used as queries to separate type II from type I kinase inhibitors.

Eventually, data collection culminated in 72,726 inhibitors of bioactivities ranging from 0.001 nM to a maximum of 1.0 mM, with lists of inhibitors ranging from 376 to 2498 for each protein kinase.

### Analysis of matched molecular pairs and activity cliffs

A pair of ligands is defined as a "matching molecular pair" (MMP) if just one chemical transformation separates them from one another^26,36^. In the current project, MMPs within inhibitors population of each protein kinase were identified using the “Find Activity Cliffs using MMPs” protocol implemented in Discovery Studio 4.5. The following settings were implemented: The maximum number of heavy atoms in the fragments that define an MMP (not including attachment points) was set to 5 atoms. MMPs with bigger fragments are not counted. Additionally, the minimum number of heavy atoms in the core that define an MMP (not including attachment points) was set to be 10 atoms. MMPs with smaller cores are not counted. MMPs with activity difference threshold exceeding 100 folds (i.e., 2 log cycles) were considered to be ACs.

Nevertheless, in order to eliminate the possibility of activity cliffs (ACs) being compound-dependent phenomena, we systematically screened all collected inhibitors to identify shared matched molecular pairs (MMPs) across different kinases. Subsequently, we evaluated their bioactivities against the corresponding protein kinases. This comprehensive analysis unveiled 24 MMPs consistently observed across different kinase targets. Among these, 19 MMPs demonstrated varying AC or non-AC behavior across different kinases, indicating that their activity cliff behaviors are independent of the specific compounds (refer to Table 3 in the “Results”), prompting their inclusion in the study. Remarkably, five out of the 24 common MMPs exclusively exhibited AC behavior, even when assessed against distinct protein kinases (Table 1, see Supporting Excel file SM0 for further details). Despite this observation, we chose not to exclude these five MMPs from our investigation, as they were tested against only two homologous kinases each. This limited testing context is insufficient to definitively conclude that their behaviors are strictly compound-dependent. Table 2 and Supporting Excel file SM1 show the collected kinases, the count of collected of inhibitors, MMPs and ACs within each set of inhibitors.

### Scanning machine learners (MLs)

In following ML experiments, the calculated protein descriptors served as explanatory variables, while the ratio ACs/MMPs (Table 2) served to define the response classes. We classified the collected kinases as follows: Kinases with no ACs were assigned to “No ACs” category, those which exhibit 0 < ACs/MMPs% ≤ 1.0% were arbitrary assigned to “Low ACs” category. Likewise, kinases with ACs/MMPs% values > 1.0% were arbitrary assigned to the “High ACs” category. The collected kinases (Table 2) were randomly divided into training and testing sets. The testing set consists of 16 proteins marked with asterisks in Table 2 (see Supporting Excel file SM2) with 154 crystallographic structures (see supporting Excel file SM3). Where, the training set comprises 64 protein (see supporting Excel file SM4) with 617 crystallographic structures (see supporting Excel file SM5). Several MLs were scanned to identify the best ML capable of correlating ACs propensity with protein properties.

ML models were evaluated based on their accuracies (Eq. 1)^37–39^ and Cohen’s Kappa values (κ, Eq. 2)^40^ in classifying the training set into “No ACs”, “Low ACs” or “High ACs” using leave-20%-out cross-validation.

1 $$Accuracy = \frac{{{\text{TP}} + {\text{TN}}}}{{\text{N}}}$$

2 $$\upkappa =\frac{Po-Pe}{1-Pe}$$

where TP and TN are the numbers of truly identified proteins as “No ACs”, “Low ACs” or “High ACs”, respectively, by the particular ML. N is the number of all observations in the training list. Pe is the probability of chance agreement (hypothetical) calculated from the probabilities of each observer randomly seeing each category in the data. Po is the relative observed accuracy (i.e., agreement among raters). If the raters (i.e., real versus predicted ACs responses) are in complete agreement, then κ = 1. If there is no agreement among the raters other than what would be expected by chance (as given by Pe), κ = 0. Negative Cohen’s kappa value implies the agreement is worse than random, while 0.01–0.20 as none to slight agreement, 0.21–0.40 as fair agreement, 0.41–0.60 as moderate agreement, 0.61–0.80 as substantial, and 0.81–1.00 as almost perfect agreement^40^.

Leave-20%-out (or fivefold cross-validation) validation involves excluding 20% of the training data points, then constructing the ML model using the remaining training data. The resulting model is subsequently used for classifying the excluded data. The procedure is repeated until all data points are excluded from the training list and predicted at least once. Accuracy and Cohen’s Kappa values are computed by comparing ML model classification results with real bioactivity classes. Conversely, calculating accuracy and Cohen’s Kappa values against the testing set involves comparing the classification results of the particular ML model with the actual classes of the testing data^24^.

### Assessed machine learners

Due to substantial variances in ProtrWeb-generated descriptors, and a notable lack of normalization in a significant portion of them, our evaluation focused on machine learning algorithms that do not strictly require data normalization. Specifically, we considered extreme gradient boosting, random forest, and Naïve Bayesian algorithms, as they are known for their resilience to feature scaling issues^41–43^. On the other hand, we chose to assess the probabilistic neural network learner node implemented in KNIME because it automatically normalizes input features^24^.

**Extreme Gradient Boosting (XGBoost):** XGBoost is a decision tree (DT)-based method that uses an ensemble of weak DT-type models to create new boosted DT-type models with a reduced loss function^44^. We implemented the XGBoost Learner node within KNIME Analytics Platform (Version 4.1.3) with the following settings: Tree booster was implemented with depth wise grow policy, boosting rounds = 100, Eta = 0.3, Gamma = 0, maximum depth = 6, minimum child weight = 1, maximum delta step = 0, subsampling rate = 1, column sampling rate by tree = 1, column sampling rate by level = 1, lambda = 1, Alpha = 0, sketch epsilon = 0.03, scaled position weight = 1. Maximum number of bins = 256, Sample type (uniform), Normalize type (tree), and Dropout rate = 0.

**Random Forest (RF):** RF is a classification multipurpose ML strategy based on Decision Trees ensembles (DTs)^45^. Each individual tree independently predicts a classification and “votes” for the related class. Majority of the votes decide the overall predictions. We implemented Weka-RF learner node within KNIME Analytics Platform (Version 4.7.5) with the following settings: Splitting criterion is the Information Gain Ratio (normalizes the standard information gain by the split entropy to overcome any unfair preference for nominal splits with many child nodes), Number of trees = 100. No limitations were imposed on the number of levels or minimum node size. The accuracy was calculated using out-of-bag internal validation.

**Naïve Bayesian (NB):** NB classifier assumes each descriptor to contribute independently to the probability that certain observation (e.g., a protein kinase) belongs to a particular class (e.g., having or not having ACs). The probability of certain observation to belong to a particular class is the multiplication of the individual probabilities of that class within each individual descriptor^46,47^. We implemented NB learner node within KNIME Analytics Platform (Version 4.7.5) with the following parameters: Default probability = 0.0001, minimum standard deviation = 0.0001, threshold standard deviation = 0.0 and maximum number of unique nominal values per attribute = 20.

**Probabilistic neural network (PNN):** Probabilistic neural network (PNN) based on the Dynamic Decay Adjustment method on labeled data using Constructive Training of Probabilistic Neural Networks as the underlying algorithm^48,49^. We implemented PNN Learner node within KNIME Analytics Platform (Version 4.7.5) using PNN theta minus = 0.2 and theta plus = 0.4 and without specifying maximum number of epochs so that the PNN process is repeated until stable rule model is achieved.

### Global feature importance (GFI)

The enormous number of protein descriptors (about 9900) suggests that any associated ML model(s) would probably encounter overfitting problems. Additionally, numerous descriptors render the process of genetic mining for optimal subset of descriptors quite challenging. Therefore, it is crucial to limit the number of descriptors of reasonable ML models (found during ML scanning) to only those that could be impactful. This was done by removing constant values and low variability descriptors (done automatically by ML KNIME nodes) followed by implementing the Global Feature Importance (GFI) KNIME node. This component identifies influential descriptors and rank them according to their importance. It requires a testing set that represents the entire distribution of the training set. GFI implements surrogate models, which are simply interpretable models that are trained to mimic the behavior of the original model by overfitting its predictions. The assumption is that if the interpretable surrogate model can accurately predict the same outcomes as the original model, it may be used to understand how the input features relate to those outcomes.

Surrogate Random Forest model was implemented in the current research. In this model, feature significance is determined by tallying the number of splits a feature has received and at what rank (level) inside the random forest trees^50^. GFI was applied against best performing MLs, namely, XGBoost^44^ and RF^45^. The resulting models (each ML with associated descriptors of GFI exceeding zero) were validated by judging their classification powers (Accuracy and Cohen’s kappa values) based on their abilities to correctly classify testing and training sets into “No ACs”, “Low ACs” or “High ACs”. The leave-20%-out cross-validation was used for the training set.

### Genetic algorithm (GA)

Successful MLs were subsequently combined with GA to select subset of descriptors, from those designated to be impactful by GFI, to construct optimal ML models.

The GA cycle is comprised of four phases^27^: (1) Encoding mechanism; (2) Determination of a fitness function; (3) Creation of a chromosomal population; (4) Genetic manipulation of chromosomes. This article implements a gene-based encoding system in which suggested models are encoded as vectors (chromosomes) and the presence or absence of descriptors in a given model is encoded by individual bins (genes), i.e., each value in the gene string representing an independent variable (0 = absent, 1 = present). An initial number of random models (chromosomes) are generated. Each chromosome has a fitness value that indicates how successful it is in comparison to other chromosomes. Genetic manipulation involves mating among successful chromosomes and mutation of some genes within randomly selected chromosomes. The GA KNIME node was implemented herein using the following parameters^27^: Population of initial random chromosomes = 100, maximum number of generations to exit from a genetic selection cycle = 500. The fitness criterion was configured to be Cohen’s Kappa value of the ML model resulting from features selected by each genetic chromosome (implementing leave-20%-out cross-validation for the training set).

### ML model evaluation using variable classification thresholds

To further assess the robustness of the successful ML models (i.e., GA-RF and GA-XGboost, see Machine Learning section in “Results”) we opted to challenge them against two distinct additional kinase classification approaches, namely, (A) A binary classification scheme in which kinases with no ACs were assigned to “No ACs” category, while those of ACs/MMPs% > 0% were assigned to the “With ACs” category (Threshold A in Table 4). (B) A ternary classification scheme in which kinases with no ACs were assigned to “No ACs” category, while those showing 0 < ACs/MMPs% ≤ 2.59% were assigned to the “Low ACs” category. However, kinases with ACs/MMPs% values > 2.59% were assigned to the “High ACs” category (Threshold B in Table 4). The value 2.59% is the average of ACs/MMPs percentage + 1.0 standard deviation calculated for the collected kinases.

**Table 4:** The best MLs models and their success statistical criteria.

ML	GA-selected features^a^	Activity cliffs definitions	Leave-20%-out cross validation using training set^b^		External testing set^c^		Average of 1000 Y-Scrambling trials based on Leave-20%-Out^d^ cross-validation of training data (maximum values in brackets)	
			Accuracy^e^	Cohen’s κ^f^	Accuracy^e^	Cohen’s κ^f^	GA feature selection^j^	GFI-Selected Features^k^
							Accuracy/Cohen’s κ	Accuracy/Cohen’s κ
GA-XGboost	VME	Default ACs Definitions^g^	0.69	0.49	0.69	0.52	0.38 (0.59)/− 0.02 (0.35)	0.38 (0.61)/− 0.03 (0.34)
	GTT							
	YDG	Threshold (A)^h^	0.80	0.59	0.81	0.61	0.50 (0.72)/− 0.02 (0.44)	0.49 (0.78)/− 0.03 (0.56)
	FTA							
	EFV	Threshold (B)^i^	0.77	0.59	0.63	0.37	0.41 (0.66)/− 0.03 (0.40)	0.41 (0.70)/− 0.03 (0.49)
	DAYM780201.lag5							
GA-RF	VME	Default ACs Definitions^g^	0.67	0.47	0.75	0.62	0.38 (0.63)/− 0.01 (0.38)	0.39 (0.56)/− 0.02 (0.29)
	GTT							
	DPS	Threshold (A)^h^	0.78	0.56	0.69	0.38	0.49 (0.70)/− 0.02 (0.41)	0.49 (0.72)/− 0.03 (0.42)
	VQH							
	EMY	Threshold (B)^i^	0.67	0.43	0.69	0.49	0.41 (0.63)/− 0.02 (0.36)	0.42 (0.63)/− 0.03 (0.34)
	CHAM820101.lag6							
	prop5.G2.residue0							

^a^Tripeptide composition defined as N_tripeptide_/(N-2), where N_tripeptide_ is count of the particular tripeptide in the protein and N is length of the protein sequence. FTA: phenylalanine, threonine and alanine; VME: valine, methionine and glutamic acid; YDG: tyrosine, aspartic acid and glycine; GTT: glycine and two threonine; EFV: glutamic acid, phenylalanine and valine; VQH: valine, glutamine and histidine; DPS: aspartic acid, proline and serine; EMY: glutamic acid, methionine and tyrosine; DAYM780201.lag5 and CHAM820101.lag6 are Moran autocorrelation descriptors and prop5.G2.residue0 is composition, transition, and distribution descriptor.

^b^Training set provided in Table 2 (supporting Excel file SM4).

^**c**^Testing set: marked with asterisks in Table 2 (supporting Excel file SM2).

^d^Y-scrambling results are shown in supporting folder 2.

^e^Accuracy: as in Eq. (1).

^f^Cohen's κ: as in Eq. (2).

^g^Kinases without any ACs classified as “No ACs” category, kinases with 0 < ACs/MMPs ≤ 1 classified as “Low ACs”, and kinases with ACs/MMPs > 1.0% classified as “High ACs”.

^h^Kinases without any ACs classified as “No ACs” category, and kinases with ACs classified as “With ACs”.

^i^Kinases without any ACs classified as “No ACs” category, kinases with 0 < ACs/MMPs ≤ 2.59% classified as “Low ACs”, and kinases with ACs/MMPs > 2.59% classified as “High ACs”.

^j^Scrambling was performed based on genetic selection of descriptors of the best models (XGboost or RF).

^k^Scrambling was performed based on impactful descriptors defined by GFI (180 descriptors).

### Y-scrambling

To validate our models and rule out chance correlations, we performed Y-scrambling^51^ using 1000 random bioactivity data generated from the training sets. Herein, the successful machine learners were challenged to create ML models using random data that were as accurate as the original nonrandomized data based on Leave-20%-Out cross-validations. We repeated Y-scrambling using GA feature selection and all impactful descriptors defined by GFI (180 features).

### Influence of binding sites on ACs propensity

To explore the potential impact of binding site properties on the likelihood of having ACs within protein kinase inhibitors, binding site exclusive descriptors were computed for the collected crystallographic complexes (771 structures) using the Proteins*Plus* online tool (https://proteins.plus)^52^. The calculated descriptors included count of hydrogen bond acceptors, count of hydrogen bond donors, count of hydrophobic moieties, count of heavy atoms (i.e., non-hydrogen atoms), count of metal ions, binding site depth (in Å), surface area (in Å^2^), volume (in Å^3^) and surface area-to-volume ratio (see supporting Excel file SM6). Subsequently, XGBoost and RF machine learners were separately coupled with genetic algorithm to select binding site descriptors that collectively exhibit best possible correlation with the propensity of having ACs. The default threshold was used as response in ML (Kinases with no ACs were assigned to “No ACs” category, those which exhibit 0 < ACs/MMPs% ≤ 1.0% were arbitrary assigned to “Low ACs” category. Likewise, kinases with ACs/MMPs% values > 1.0% were arbitrary assigned to the “High ACs” category). The resulting ML models were evaluated based on their accuracies (Eq. 1)^37–39^ and Cohen’s Kappa values (κ, Eq. 2)^40^ in classifying the training (using leave-20%-out cross-validation) and testing sets.

## Results

While earlier efforts related to ACs focused on the structural characteristics of MMPs to explain the phenomenon^1,4–6,8,9,11,12,15,26,35^, instances where MMPs exhibit ACs behavior upon interacting with some protein kinases while maintaining similar affinities with others led us to perceive ACs as protein-related phenomenon. Table 3 provides examples of closely analogous compounds (MMPs) exhibiting distinct bioactivity behaviors among various protein kinases. For instance, pair 1 in Table 3 exemplifies a single substituent variation, wherein the hydrogen atom is replaced with a fluorine atom. This alteration leads to notable differences in affinity against two kinases, namely, FLT3 and KIT, with potency varying by at least 100-folds. However, the same pair exhibits similar affinities (i.e., does not demonstrate ACs behavior) towards eight different protein kinases, specifically ABL1, CSF1R, FLT1, FYN, KDR, LCK, LYN, and SRC (refer to supporting Excel file SM0 for additional details in this regard).

### Data collection

Although activity cliffs can be artificially introduced for any protein target (e.g., by introducing an extra methylene group to a tightly fitting ligand or by replacing a deeply buried hydrogen atom with a strongly hydrophilic moiety like sulfonate), it can be safely assumed that such artefacts are not found in the ChEMBL database. This is because compounds deposited in ChEMBL are not random structures; rather they were deliberately designed by medicinal chemists in such a way to avoid loss of bioactivity. Consequently, the protein kinases we identified as cliff forming are, in reality, kinases that have the unique ability to deceive human medicinal chemists into generating activity cliffs.

However, it is still essential to collect accurate ligand binding data to successfully assess any potential relationship between protein characteristics and the propensity of having ACs. Therefore, in order to minimize the impact of inter-laboratory differences frequently present with bioactivity indicators (e.g., IC_50_), we only included inhibitors whose bioactivities were reported as Ki values^53^. Moreover, we limited ourselves to type I protein kinase inhibitors and excluded type II kinase inhibitors. This was performed by fitting the collected inhibitors against signature fragments identified for type II kinase inhibitors^35^. Inhibitors with matching fragments were excluded. ACs were defined as MMPs of bioactivity difference exceeding 100 folds.

It can be argued that the presence or absence of ACs can be a function of the explored chemical space of the particular protein kinase. For example, absence of ACs for a particular protein kinase is due to limited medicinal chemistry exploration rather than intrinsic factors associated with the protein kinase itself. Furthermore, certain ACs may be published in IC_50_ format, while others may not be reported at all since the researchers simply did not bother to measure Ki or IC_50_ for the inactive AC members after noticing their low % inhibition at a certain inhibitor concentration.

Therefore, we took two steps to address these issues. Firstly, we only collected protein kinases that have large number of reported ligands, in particular MMPs. We considered ACK1 (Table 2) as baseline threshold to include or exclude any protein kinase in the study. ACK1 has one reported AC among 54 MMPs (originating from 376 reported inhibitors), which is the least count of MMPs among all protein kinases reported to exhibit ACs (based on our data collection rules, e.g., Ki data only). As a result, it is reasonable to assume that if the binding space of a specific protein kinase has been explored by more than 54 MMPs without identifying any ACs, then this target is likely to be resistant to the ACs phenomenon. Based on this reasonable assumption, we gathered protein kinases of at least 54 known MMPs. Above this limit, the presence of even a single AC indicates that the particular protein kinase is susceptible to AC phenomenon. Still, it would be unfair to assume that all proteins in this category behave similarly; a kinase showing numerous ACs among its MMPs should differ significantly from another kinase displaying rare ACs despite having an equivalent number of MMPs.

Therefore, to deal with this dilemma we initiated our modeling endeavors by attempting regression-based machine learning to establish a correlation between the normalized counts of ACs, i.e., the ratio of ACs-to-MMPs, with protein properties. Regrettably, all our efforts in this direction proved unsuccessful, as indicated by numerous unpublished ML trials, which we believe is due to limited data.

This prompted us to take our second step in tackling the challenges presented by ACs-related data limitations, namely, by transitioning to classification-based machine learning. In this direction, we categorized the collected kinases into three classes based on their ACs population: Kinases without any ACs were designated to the “No ACs” category, those with an ACs/MMPs ratio percentage between 0 and 1.0% were arbitrary placed in the “Low ACs” category, and kinases with an ACs/MMPs percentage of > 1.0% were grouped in the “High ACs” category.

**Figure 1:**
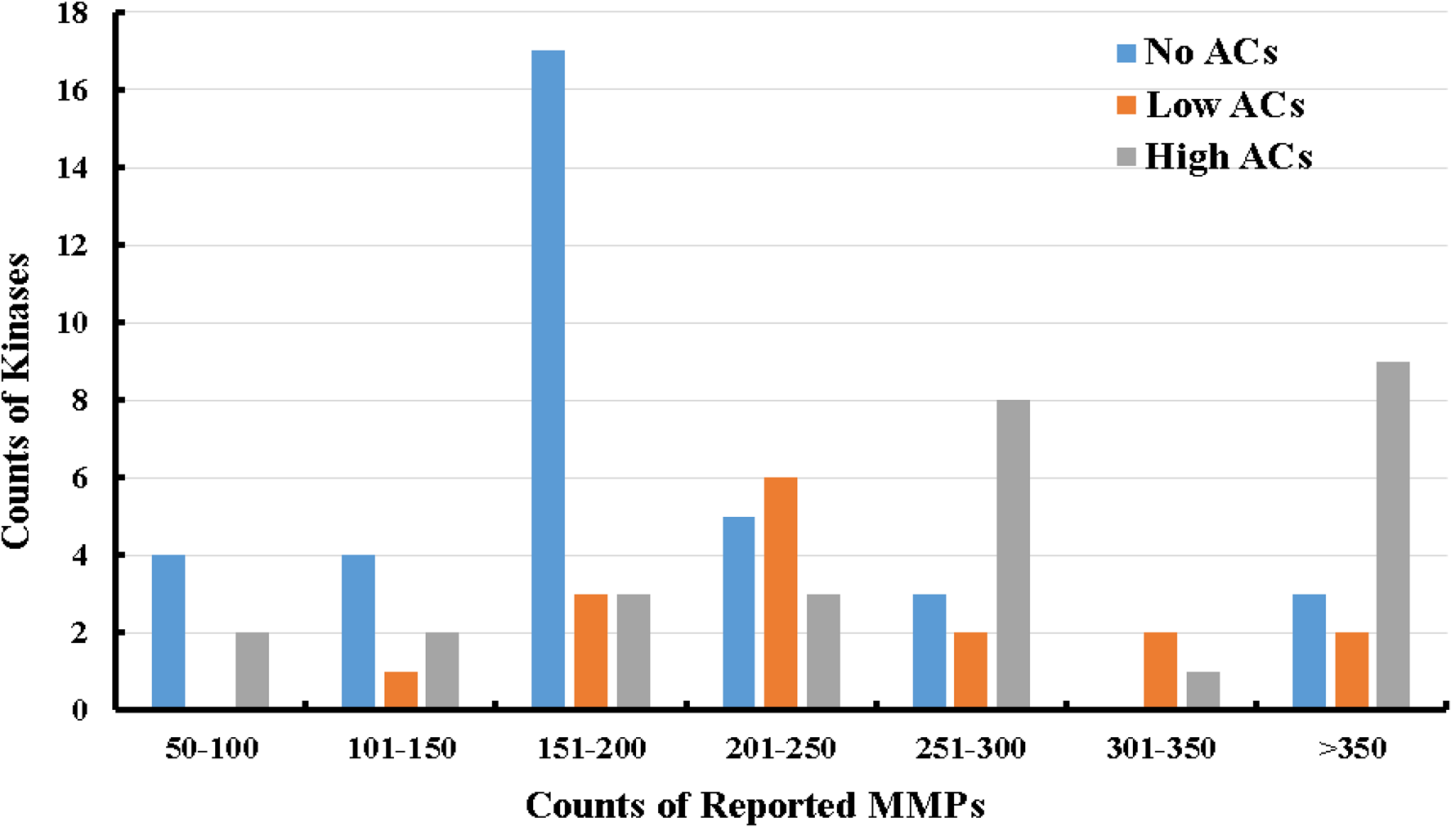
Counts of protein kinases in different classes as function to the count of reported MMPs in ChEMBL database.

**Figure 2:**
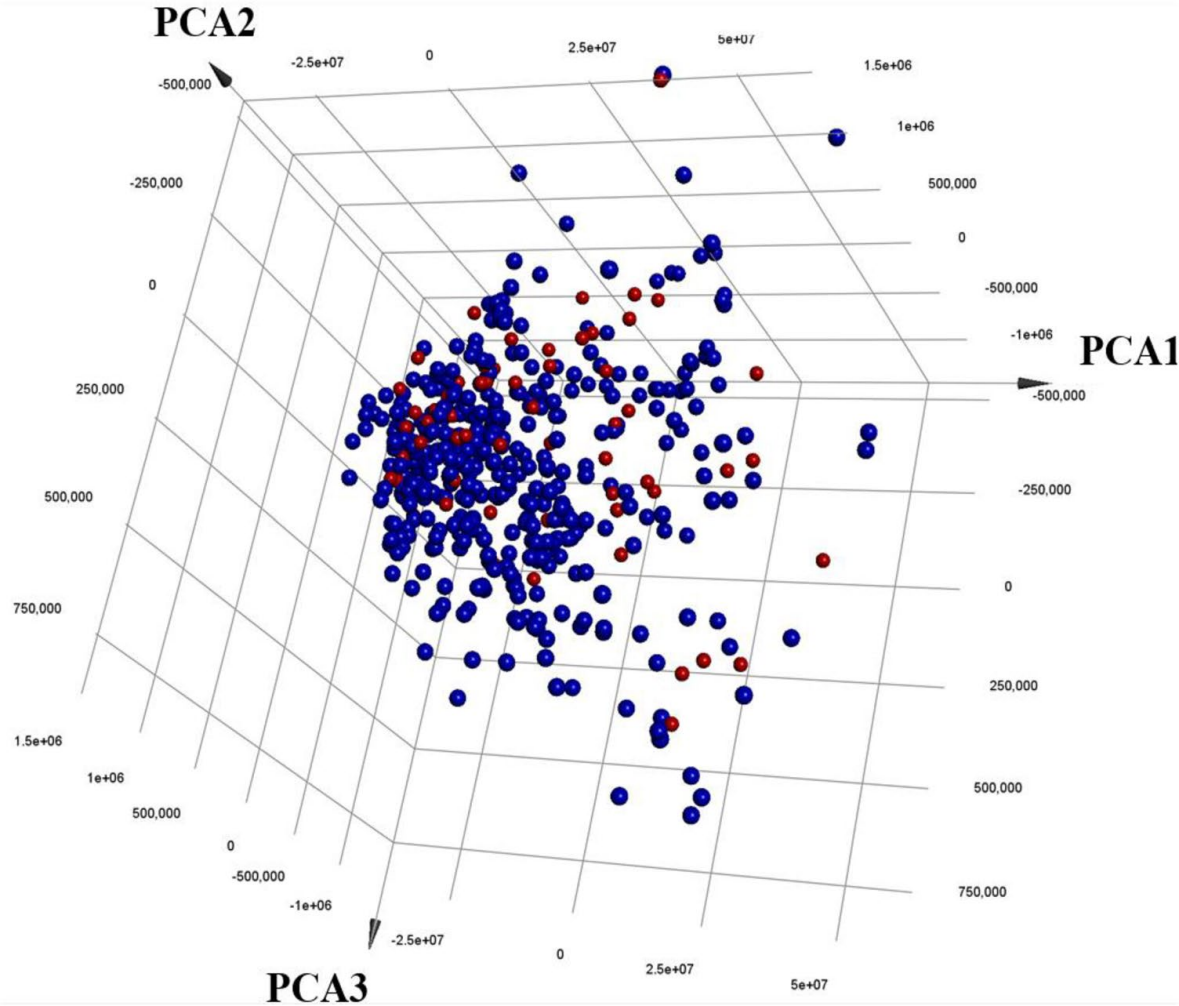
Three-dimensional plot of the top three principal components calculated based on Protr 9920 descriptors for the collected modelled list of protein kinases (red filled circles) compared to 509 known protein kinases (black filled circles).

Figure 1 shows the counts of "No ACs", "Low ACs" and "High ACs" protein kinases as function of their reported MMPs in ChEMBL database. Clearly, from the graph the “No ACs” category supersedes the "Low ACs" and "High ACs" categories in the first three intervals, i.e., 50–200. However, although protein kinases of higher MMPs counts (> 200) incline towards the ACs-vulnerable classes, still significant “No ACs” minority exists within these categories, which emphasizes the existence of AC-resistant protein kinases despite extensive medicinal chemistry exploration. Conclusions from Fig. 2 provided impetus for our proposition that the existence/absence of ACs, within certain protein kinase binders, points to the level of resistance/vulnerability of that target to ACs regardless to the extent of explored chemical space. Therefore, our use of the terms "No ACs", "Low ACs" and "High ACs" are very plausible surrogates for AC resistance/vulnerability, and should limit errors resulting from data restrictions due to the limited number of collected protein kinases, excluding IC_50_ values, or total absence of bioactivity data covering the inactive AC members as mentioned earlier. In other words, members of the “No ACs” category will remain to be considered resistant to ACs even if future extensive medicinal chemistry exploration unveils few ACs or if we overstepped some of their ACs because the corresponding bioactivities data were expressed as IC_50_ values, etc.

On the proteins side, because all protein kinases have comparable ATP catalytic sites that are frequently targeted by customized inhibitors, we hypothesize that the proclivity to have ACs is related to the entire protein matrix, not just the binding site^23^. Accordingly, complete FASTA codes of the selected targets were downloaded from UniProt (https://www.uniprot.org/). ProtrWeb (http://protr.org/) was used to generate fairly large number of diverse structural and physicochemical descriptors for the collected proteins^28^. However, due to the limited number of collected protein kinases (only 80), we opted to augment them by multiple crystallographic structures for each protein target. Corresponding three-dimensional (3D) descriptors were included as additional explanatory descriptors. This should also be valuable in evaluating the effects of 3D descriptors extracted from crystallographic structures on the ACs phenomenon: Numerous crystallographic ligand–protein structures should help to determine whether the magnitude of protein conformational shifts upon binding ligand correlates with the likelihood of encountering ACs within the corresponding ligand population. Such conformational modifications can be easily enrolled in machine learning models represented by counts of hydrogen bonds, electrostatic interactions, van der Waals forces, and π–π stacking interactions within the protein matrix. Overall, the study included 771 protein crystallographic structures representing 80 protein kinases.

Despite their limited number, the collected kinases are good representatives of the population of known protein kinases (508 members) as in Fig. 2. Clearly, the collected kinases uniformly mingle within the population of protein kinases in a 3D plot of the three main principal components calculated based on ProtrWeb descriptors (*ca.* 9900 descriptors).

### Machine learning (ML)

We subsequently applied ML to evaluate how relevant protein descriptors to the propensity of having ACs. Classification ML studies commenced by splitting the collected kinases into training and testing sets (as in Table 2, testing compounds are marked with asterisks). The training list included 617 crystallographic complexes (corresponding to 64 protein kinases, equivalent to *ca.* 80% of the collected protein kinases list) of which 238 are labeled as “No ACs”, 101 are labeled as “Low ACs” and 278 are labeled as “High ACs”. The testing set, on the other hand, included 154 complexes (corresponding to 16 protein kinases, equivalent to *ca.* 20% of the collected protein kinases list, marked with asterisks in Table 2) of which 45 are labeled as “No ACs”, 52 labeled as “Low ACs” and 57 labeled as “High ACs”. Supporting Excel files SM3 and Supporting Excel files SM5 show all details of the training and testing sets.

Subsequently, several machine learners were scanned to identify which can best correlate protein properties with the tendency of having ACs. All protein descriptors were incorporated in this stage including three-dimensional descriptors extracted from crystallographic structures. Two prominent learners emerged from this tournament, namely, XGBoost (XGB) and Random Forest (RF).

RF is a supervised ML method composed of combination of uncorrelated decision tree (DT) predictors whose prediction by committee is more accurate than that of any individual DTs^45^. XGBoost, on the other hand, is a tree-based standardized ensemble method that uses an ensemble of weak DT-type models to create new subsequent boosted DT-type models with a reduced loss function^44^.

However, to avoid overfitting due to the large number of protein descriptors (*ca.* 9,900), we opted to implement the Global Feature Importance (GFI) KNIME node to identify influential descriptors and rank them according to their significance vis-à-vis propensity of having ACs. GFI assessment identified 180 features of global importance values exceeding zero for both models (feature significance is computed by calculating the number of times and level at which a feature was selected for a split among all available features in a surrogate RF model). Expectedly, dimensionality reduction using GFI (from 9928 to 180 descriptors) enhanced the predictive power of XGBoost and RF learners against the testing set (see supporting Excel file SM7 for more details).

Remarkably, only amino acid sequence-dependent, i.e., Protr-generated, descriptors emerged as impactful in the GFI analysis, while their 3D counterparts failed to do so and were totally excluded. Still, we took an additional step to exclude the possibility that this failure is because of the limited number of 3D descriptors (8 including 4 reversible binding intra-protein interactions and their normalized forms) compared to Protr-generated descriptors (~ 9920): We reduced the dimensionality of ProtrWeb descriptors to ten latent variables using PCA. Subsequently, we re-assessed the GFI of the 18 descriptors (i.e., 10 Protr-based PCA-descriptors and 8 3D-descriptors). The details can be found in supporting Excel file SM8. Significantly, nine of the Protr latent variables ranked above their 3D counterparts. This behavior suggests that the protein sequence is essentially the main player in the propensity of having ACs rather than any protein conformational rearrangements induced upon ligand binding (encoded in the 3D crystallographic structures of the proteins). Moreover, the poor impact of the 3D descriptors suggests that adding the crystallographic structures served only to augment the datapoints by repetition, which might cause unforeseen biases within the training data. Therefore, we decided to carry out subsequent machine learning steps using the original unaugmented training and testing sets (i.e., 80 protein targets divided into 64 and 16 training and testing observations, respectively, see supporting Excel files SM2 and supporting Excel files SM4).

Despite the ability of GFI-embedded RF to rank features based on their impact on response, its capacity to identify feature interactions diminishes with a larger number of features^54^. Furthermore, the commonly used Permutation Feature Importance (PFI) metric in RF models has limitations when dealing with strongly correlated features^55^. Therefore, we opted to engage GFI-filtered descriptors in genetic algorithm (GA) feature-selection tournaments to identify the most impactful set of protein properties that influence ACs propensity. Incidentally, we refrained from implementing PCA-based latent variables in subsequent ML modeling due to their ambiguous inferences.

Table 4 shows the resulting models, their descriptors and statistical criteria. Clearly, GA-XGboost and GA-RF achieved significant accuracies and Cohen’s Kappa values upon GA-driven feature reduction. The Cohen’s Kappa values of both models ranged from 0.47 to 0.62 against training (Leave-20%-Out) and testing sets (Table 4, default ACs definitions) indicating moderate to substantial reliability^56,57^. Nonetheless, the two models fell short of perfect reliability (i.e., κ from 0.81 to 1.0)^40,56,57^, implying the existence of additional factors contributing to the ACs phenomenon, e.g., trapping a water molecule in the binding site, the ligand being a bit too big for a tight pocket, or weakening a crucial protein–ligand interaction. Another interesting inference from Table 4 is the apparent orthogonality of the two ML models, as can be deduced from their differing descriptors, suggesting the possibility of stacking the two ML models in a meta-learning model (e.g., consensus voting)^58^.

However, in our definition of ACs, we relied on Ki values to mitigate the inter-laboratory variabilities commonly observed in IC_50_ values. This approach may have overlooked some information pertaining to ACs associated with IC_50_ data. Consequently, it is possible that certain kinases, initially labeled with a low number of ACs, may indeed have more ACs reported in IC_50_ format. Furthermore, protein kinases initially categorized with a low number of ACs might exhibit additional ACs in future research, potentially transitioning from the “Low ACs” to the “High ACs” class. Considering these possibilities, we chose to assess our models using an extra two-class response: “With ACs” and “Without ACs”. Similarly, to account for the potential scenario where certain protein kinase members labeled as “High ACs”, in our default ACs classification, could gain even more ACs in future research, leading to a division within this category between those with significantly more ACs and those closer to the “Low ACs” category, we deemed it reasonable to expand the “Low ACs” category. Therefore, we thought it is reasonable to expand the “Low ACs” category to include such members using the concept of “mean plus one standard deviation”. Needless to say, this concept is used in statistical analysis to identify values that are significantly different from the average^59^.

Therefore, as additional validation of our optimal GA-ML models we decided to evaluate them on the basis of two additional alternative ACs definitions (i.e., ACs thresholds), namely, (A) A binary classification in which kinases with no ACs were assigned to “No ACs” category, while those of ACs/MMPs% > 0% were assigned to “With ACs” category (Threshold A in Table 4). (B) A ternary classification in which kinases with no ACs were assigned to “No ACs” category, while those showing ACs were further divided into two groups based on the average of their ACs/MMPs% values plus one standard deviation (equals 2.59%). Therefore, kinases exhibiting 0 < ACs/MMPs% ≤ 2.59% were assigned to the “Low ACs” category. While, kinases of ACs/MMPs% values > 2.59% were assumed to be significantly different from the mean, and therefore, were assigned to a distinct “High ACs” category (Threshold B in Table 4). Clearly, from Table 4, both optimal ML models (GA-RF and GA-XGboost) maintained successful statistical criteria against training and testing sets despite varying ACs class definitions. Noticeably, both optimal models demonstrated successful statistical performance on both training and testing sets, despite variations in ACs class definitions. However, GA-XGboost exhibited the best performance when utilizing the binary kinase classification (Threshold A, Table 4). On the other hand, the GA-RF model showed better performance in the ternary classification system (Threshold B, Table 4) compared to its performance in the binary classification system (Threshold A). This trend suggests that GA-XGboost and GA-RF are complementary models, and using them together should enhance the prediction accuracy of ACs propensities among kinases.

To rule out the possibility of chance correlations, we opted to validate our models using Y-scrambling^51^. 1000 random bioactivity data were created from the training sets. Then, the successful machine learners were challenged to create ML models using random data as accurate as the original nonrandomized data based on Leave-20%-Out cross-validations. We repeated Y-scrambling based on (1) GA feature selection, and (2) all impactful descriptors defined by GFI (180 features). The results of 1000 Y-scrambling trials are summarized in Table 4, while the detailed results can be found in supporting folder 2. Notably, the nonrandomized training sets unanimously yielded models of higher leave-20%-out cross-validation accuracies and Cohen's Kappa values compared to all their corresponding randomized experiments (whether GA-selected or impactful GFI descriptors). Overall, these findings strongly emphasize the validity of the two machine learning models.

Although kinase-binding sites are generally conserved to bind to ATP, we opted to investigate any potential role played by binding sites on the ACs phenomenon. Towards this, we explored the possibility of correlating binding site exclusive properties with the propensity of ACs through ML. However, given that kinase-binding sites consist of non-continuous amino acid sequences originating from distinct parts of the protein chain, it is inappropriate to extract Protr-based descriptors for binding sites. This is because the Protr package necessitates continuous, uninterrupted amino acid sequences for proteins under assessment [28]. As an alternative, we chose to extract 3D properties reflecting the pharmacophoric characteristics of the binding sites of the collected crystallographic complexes (771 structures) using the Proteins*Plus* online tool (https://proteins.plus, see the experimental section Influence of Binding Sites on ACs Propensity) [52]. The computed descriptors were then utilized to search for optimal ML models employing the best-performing machine learners in our study, namely XGBoost and RF, coupled with genetic algorithm. The resulting models and their success criteria are summarized in Table 5. Clearly, the best possible ML models failed to correlate exclusive binding site properties with the likelihood of having ACs.

**Table 5:** The best possible ML models connecting binding site properties with ACs propensity at default ACs Definition.

ML	GA-selected features^a^	Training set^b^ leave-20%-out cross validation		External testing set^b^	
		Accuracy^c^	Cohen’s κ^d^	Accuracy^c^	Cohen’s κ^d^
GA-XGboost	Acceptors	0.46	0.12	0.42	0.11
	Depth (Å)				
	Hydrophobicity				
	Heavy Atoms				
	Volume (Å^3^)				
GA-RF	Acceptors	0.50	0.15	0.44	0.14
	Depth (Å)				
	Hydrophobicity				
	Heavy Atoms				
	Surface (Å^2^)				
	Volume (Å^3^)				

^a^Acceptors: Count of hydrogen bond acceptor within the binding pocket, Depth: the depth of the binding pocket in Å extending from outer rim to the furthest point in the biding site, Hydrophobicity: count of hydrophobic amino acids in the binding pocket, Heavy Atoms: sum of the non-hydrogen atoms within the binding pocket, Volume: the volume of the binding pocket in Å^3^, Surface: the surface area of the binding pocket in Å^2^.

^b^Training set and Testing set are provided in Supporting File SM6.

^c^Accuracy: as in Eq. (1).

^d^Cohen's κ: as in Eq. (2).

## Discussion

Unsurprisingly, all our attempts to exclusively correlate the ACs phenomenon with kinase-binding sites failed to reveal any feasible relationship. This conclusion is substantiated by experimental evidence.

**Figure 3:**
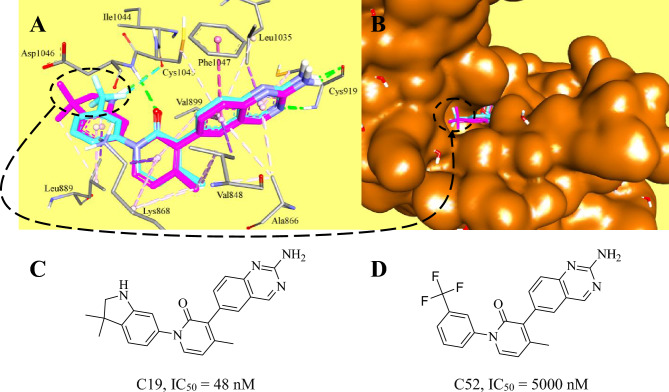
Crystallographic structures of KDR co-crystallized with AC pair (PDB codes: 3CP9 and 3CPC). (**A**) Superimposi tion of complexed ligands C19 (purple) and C52 (cyan) within KDR showing binding interactions anchoring the bound ligands, H-bonds are shown as green dotted lines, hydrophobic and π-stacking interactions are shown as pink and light pink dotted lines. (**B**) Water-accessible surface (Brown, Connelly’s Surface) covering KDR protein complexed with superimposed AC pair. (**C**, **D**) Chemical structure of C19 and C52.

For instance, Fig. 3 illustrates crystallographic complexes corresponding to an AC pair bound within the protein kinase KDR (PDB codes 3CP9 and 3CPC). Both ligands are evidently closely aligned within the binding pocket and are anchored via identical binding interactions. Notably, the two compounds exhibit only slight differences at the binding site's orifice, located distantly from their primary binding interactions within KDR’s binding site, as depicted in Fig. 3B. Remarkably, the binding pocket maintains the same conformational state upon binding to both ligands, indicating minimal involvement of the binding pocket in the ACs phenomenon.

These conclusions underscore the entire protein matrix as the fundamental factor in controlling this phenomenon. Conversely, while relying on machine learning-selected protein descriptors to extract deep insights into the mechanisms underlying ACs may not be very satisfactory, machine learning remains feasible to draw some inferences about the protein factors that affect the formation of ACs. Clearly, from Table 4, the best GA-ML models reveal that certain tripeptide sequences apparently play certain role in the propensity of ACs. Notably, the tripeptide sequences **FTA** (phenylalanine, threonine, and alanine), **VME** (valine, methionine, and glutamic acid), **YDG** (tyrosine, aspartic acid, and glycine), **GTT** (glycine and two threonine), **EFV** (glutamic acid, phenylalanine, and valine), **VQH** (valine, glutamine, and histidine), **DPS** (aspartic acid, proline, and serine), and **EMY** (glutamic acid, methionine, and tyrosine) are of particular significance. Likewise, emergence of the Moran autocorrelation descriptors **DAYM780201.lag5** (relative mutability index) and **CHAM820101.lag6** (a polarizability parameter), as well as the composition, transition, and distribution descriptor **prop5.G2.residue0** in the optimal GA-ML models suggests significant influence of inherent properties of the entire protein on the likelihood of the occurrence of ACs^28,54,55,60–62^.

Table 6 illustrates how these descriptors vary across different kinase classes (defined by our default ACs definitions). The **VME** tripeptide composition descriptor scored highest frequency of appearance (and average value) among “No ACs” class compared to the other two classes where it only emerged marginally. **YDG** tripeptide composition shows similar trend as it consistently decreased upon moving from “No ACs” to “Low ACs” and “High ACs”. Likewise, **VQH** tripeptide composition is totally absent from the “High ACs” category. On the contrary, the **GTT** tripeptide composition is absent in the “No ACs” category but shows an escalating appearance in the “Low ACs” and “High ACs” classes with frequencies of 6.3% and 21.4%, respectively. The corresponding averages of the **GTT** tripeptide composition follows a similar trend. Likewise, the **EMY** tripeptide composition exhibits a consistent increase as we move from the "No ACs" to the "Low ACs" and "High ACs" categories.

**Table 6:** Descriptor variabilities among collected kinases^a^.

Descriptors	Kinases in the “No ACs” Class				Kinases in the “Low ACs” Class				Kinases in the “High ACs” Class			
	%Kinases^b^		Average^c^	SD^d^	%Kinases		Average	SD	%Kinases		Average	SD
VME	52.8	8.26 × 10^−4^		1.03 × 10^−3^	6.3	1.28 × 10^−4^		5.11 × 10^−4^	7.1	6.52 × 10^−5^		2.41 × 10^−4^
YDG	16.7	2.18 × 10^−4^		5.26 × 10^−4^	12.5	1.87 × 10^−4^		5.15 × 10^−4^	3.6	2.96 × 10^−5^		1.56 × 10^−4^
VQH	5.6	1.04 × 10^−4^		4.60 × 10^−4^	25.0	3.13 × 10^−4^		5.98 × 10^−4^	0.0	0.0		0.0
GTT	0.0	0.0		0.0	6.3	4.50 × 10^−5^		1.80 × 10^−4^	21.4	4.12 × 10^−4^		9.65 × 10^−4^
EMY	2.8	4.72 × 10^−5^		2.83 × 10^−4^	6.3	9.51 × 10^−5^		3.81 × 10^−4^	14.3	1.22 × 10^−4^		3.07 × 10^−4^
EFV	13.9	2.14 × 10^−4^		6.24 × 10^−4^	12.5	2.13 × 10^−4^		6.11 × 10^−4^	10.7	8.75 × 10^−5^		2.58 × 10^−4^
FTA	5.6	1.21_X_10^−4^		5.05 × 10^−4^	25.0	3.70 × 10^−4^		7.10 × 10^−4^	7.1	1.25 × 10^−4^		4.74 × 10^−4^
DPS	22.2	2.26 × 10^−4^		4.95 × 10^−4^	31.3	5.41 × 10^−4^		8.88 × 10^−4^	3.6	2.96 × 10^−5^		1.56 × 10^−4^
DAYM780201.lag5	NA^e^	1.35 × 10^−2^		4.59 × 10^−2^	NA	1.63 × 10^−2^		4.42 × 10^−2^	NA	− 4.16 × 10^−3^		4.04 × 10^−2^
CHAM820101.lag6	NA	4.56 × 10^−2^		4.98 × 10^−2^	NA	1.30 × 10^−2^		4.78 × 10^−2^	NA	4.02 × 10^−2^		4.54 × 10^−2^
Prop5.G2.residue0	NA	1.60 × 10^−1^		6.89 × 10^−2^	NA	1.56 × 10^−1^		5.13 × 10^−2^	NA	1.65 × 10^−1^		8.28 × 10^−2^

^a^Classes as demarcated by the default ACs definitions.

^b^The percentage of kinases in the corresponding category that have the specific tripeptide at least once in their amino acid sequences.

^c^Average of tripeptide composition within the particular kinase class. For how this is calculated see footnote of Table 4.

^d^Standard deviation of tripeptide composition among the particular kinase class.

^e^NA: Not Applicable.

Notably, Table 4 indicates that the tripeptide compositions **VME** and **GTT** are particularly prominent in both optimal models, highlighting their significance. Unquestionably, when two distinct machine-learning models concur on particular shared features, it significantly strengthens the potential significance of these features. Table 6 further supports this finding, where these two descriptors show a clear correlation with ACs-related kinase classes.

However, the behaviors of other GA-ML selected descriptors, including the tripeptide compositions **EFV**, **FTA**, and **DPS**, as well as the Moran autocorrelation descriptors **DAYM780201.lag5** and **CHAM820101.lag6**, and the composition, transition, and distribution descriptor **prop5.G2.residue0**, do not show a clear correlation with the ACs propensity. Nevertheless, their appearance in the optimal ML models indicates certain complex roles played by these descriptors in the ACs phenomenon.

To delve further into the role these specific tripeptides play in the ACs phenomenon, we sought to address several inquiries: Where within the protein kinases do these tripeptides reside? Do they exhibit greater prevalence within specific kinase subfamilies? Are these motifs associated with particular functional or structural motifs that could elucidate the observed outcomes? To address these inquiries, we compiled comprehensive information regarding the families and subfamilies of the collected protein kinases, along with the specific domains where each of the tripeptides is situated within their corresponding kinases. The collected information is shown in supporting excel file SM9. The data suggests that there is no discernible trend in the distribution of these tripeptides among specific protein kinase families or subfamilies. However, approximately 52% of these tripeptides belong to the protein kinase domains of the collected proteins. Still, the remaining 48% are dispersed across various domains without exhibiting any discernible pattern. Overall, based on this information, it is challenging to draw definitive conclusions regarding how these specific tripeptide sequences influence the ACs phenomenon. Nevertheless, as pioneers in proposing the protein-related nature of the AC phenomenon, we hypothesized in earlier publications that the presence of potent AC twin members induces substantial, entropy-driven conformational changes in the target protein^24,25^. This hypothesis finds support in experimental evidence. For instance, the closely related potent analogs outlined in Table 7, which bear structural resemblances to AC pairs, display markedly distinct entropy-enthalpy binding thermodynamics^63^. Furthermore, they exert significantly disparate effects on the conformation of the entire target protein: the entropic binder (the lower entry in Table 7) induces substantial conformational rearrangements, whereas its enthalpic counterpart (the upper entry in Table 7) elicits only subtle modifications in protein structure.

**Table 7:** Inhibition constants and thermodynamic data of two close analogues binding to homodimeric tRNA-binding protein^63^.

Compound	PDB code	ΔG [KJ/mol]	ΔH [KJ/mol]	TΔS [KJ/mol]	Protein distortion upon binding
	4LEQ	− 37.9 ± 0.4	− 40.2 ± 2.2	2.4 ± 2.1	Limited (or no) conformational modifications
	4KWO	No detectible heat signal in ITC experiment (ΔH ≈ 0)		≈ 40	Introduces huge conformational rearrangements of the protein

Consequently, we posit that the tripeptide sequences identified by the optimal GA-ML models listed in Table 4, are intricately linked to the inherent predisposition of a specific protein kinase to undergo substantial conformational alterations driven by entropic binding. Yet, quantifying these entropic disparities poses a significant challenge due to the intricate nature of protein conformational changes induced by entropy-driven ligand binding^63–67^, necessitating molecular dynamics simulations spanning tens of microseconds^68^.

## Conclusion

Our study investigated ACs in the context of protein kinases, crucial therapeutic targets in drug discovery. We proposed that the presence of ACs depends on the specific target protein and its complete structural context, extending beyond the binding site alone. Our findings highlighted specific tripeptide sequences, such as FTA, VME, YDG, GTT, EFV, VQH, DPS, and EMY, as determinants of the propensity of ACs. Additionally, descriptors related to the overall protein's inherent properties, like Moran autocorrelation and composition, transition, and distribution descriptors, were also significant in influencing ACs. Overall, our work sheds light on the intricate interplay between protein properties and AC occurrence, with potential implications for drug discovery and design. Future research in this area could deepen our understanding of the underlying mechanisms of ACs.

### Supplementary Information

Supplementary Information.

## Data Availability

The data generated or analyzed during this study are available within the article and its supporting material.
