# Supplementary material for: Protein characteristics substantially influence the propensity of activity cliffs among kinase inhibitors
Source: Sci Rep. 2024 Apr 20;14:9058. doi: 10.1038/s41598-024-59501-w (PMC11032345; doi:10.1038/s41598-024-59501-w)
Supplement: Supplementary file 1 — Supplementary Information. [file 41598_2024_59501_MOESM1_ESM.zip › Supporting_revised/Supporting folder 1_Knime Workflows/Screenshots for workflows.docx]

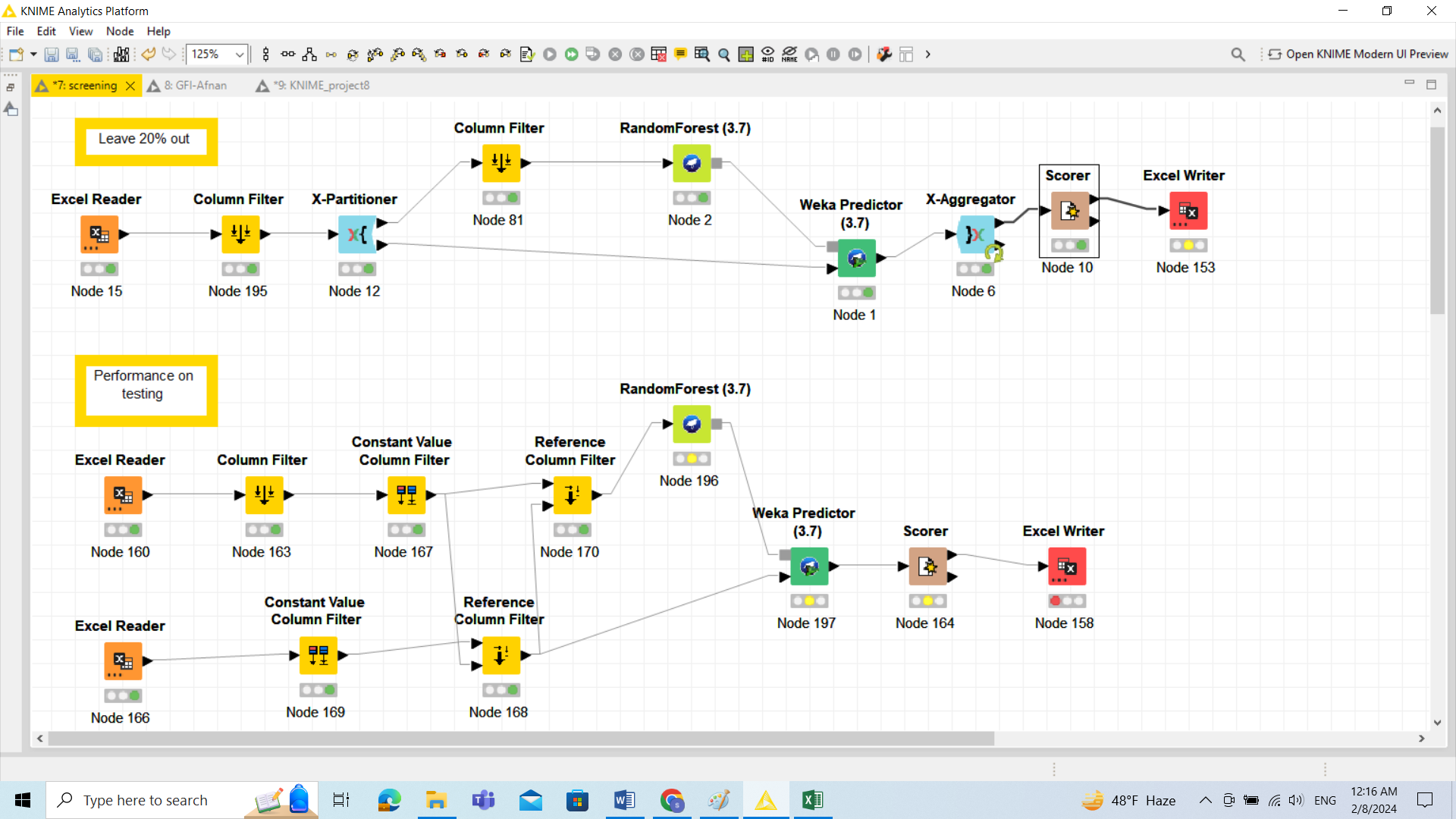


**Figure S1.** Screening workflow using random forest.


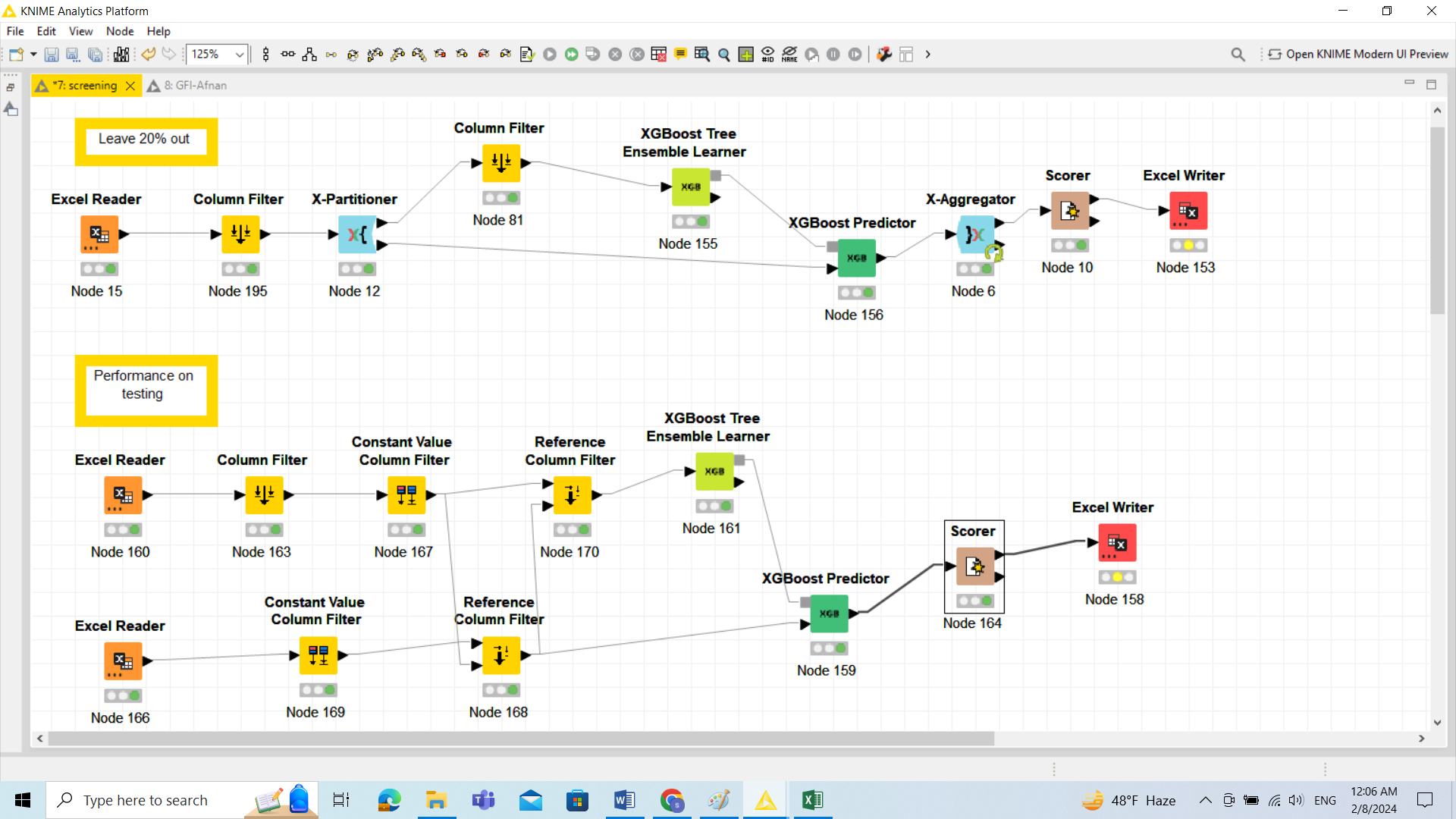


**Figure S2.** Screening workflow using XGBoost.


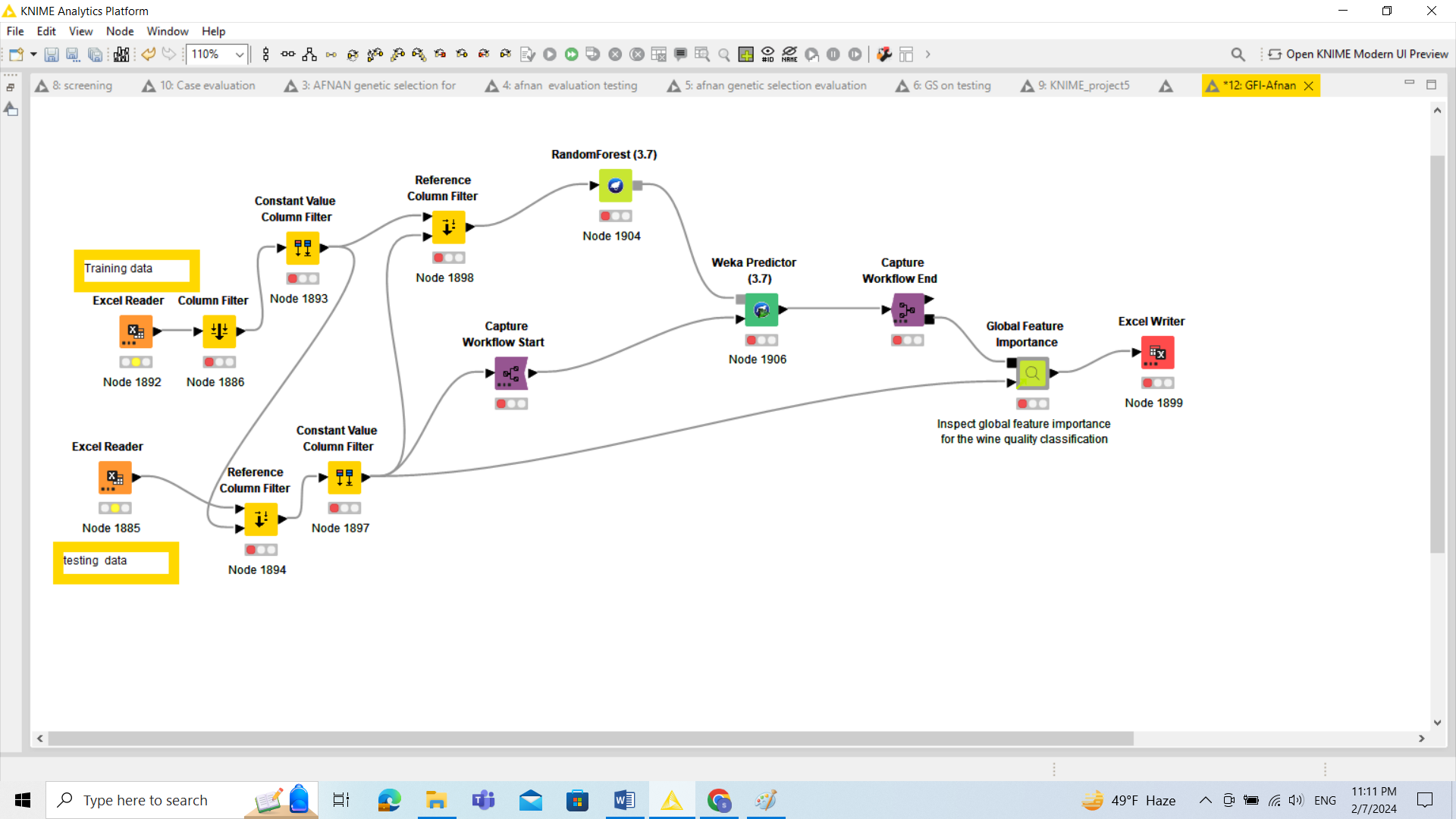


**Figure S3.** GFI workflow using random forest.


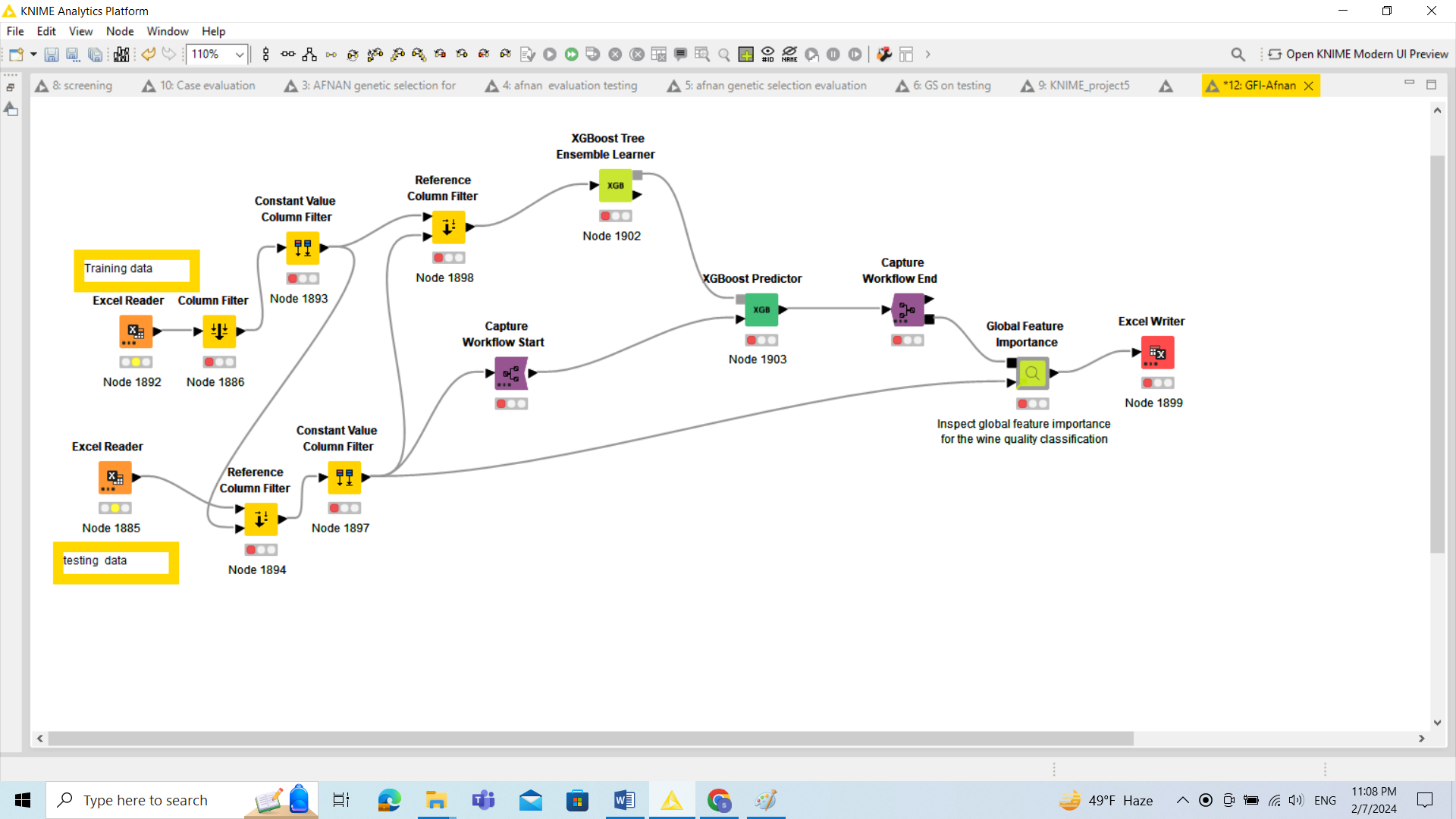


**Figure S4.** GFI workflow using XGBoost.


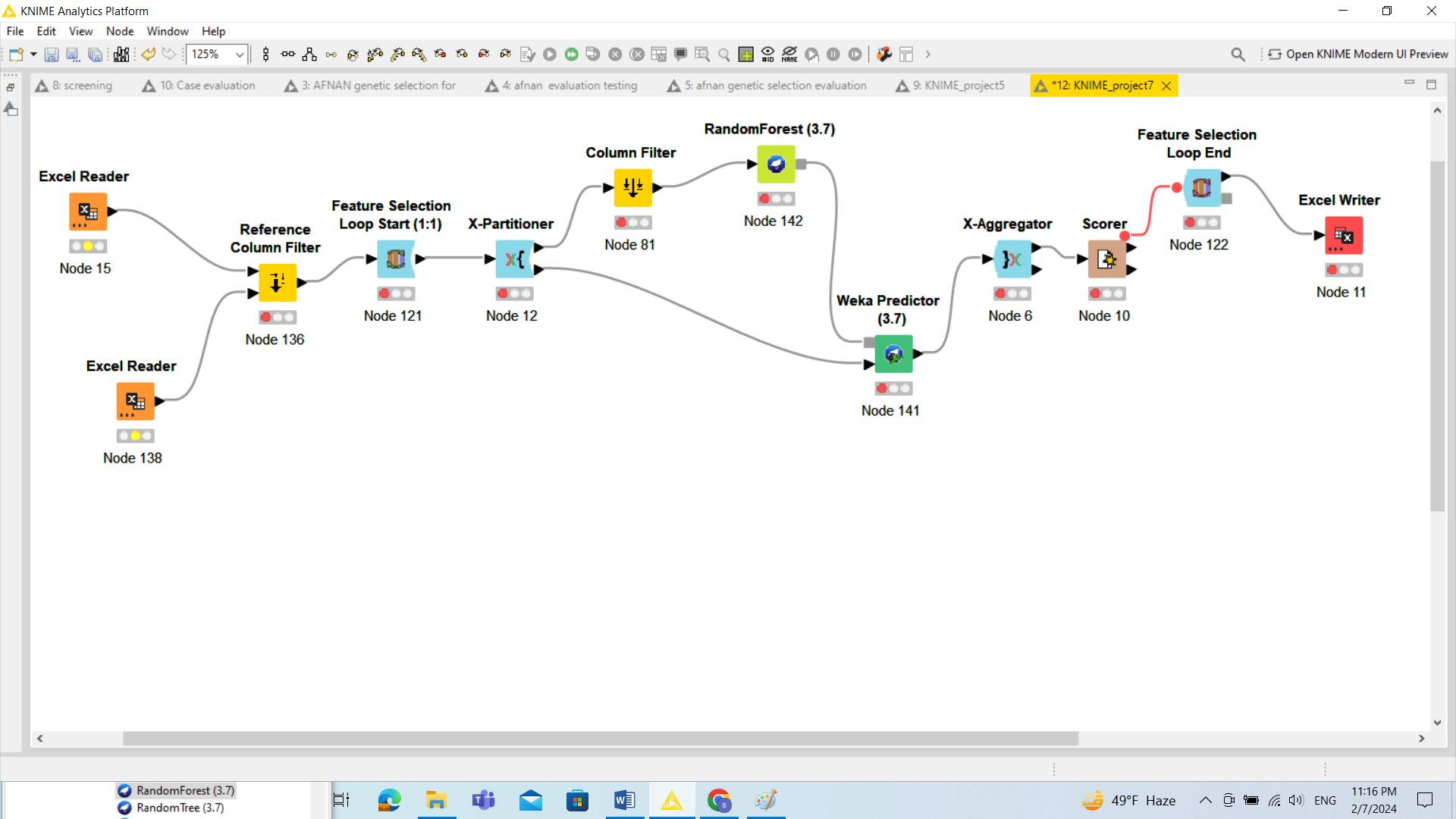


**Figure S5.** Genetic function workflow using random forest.


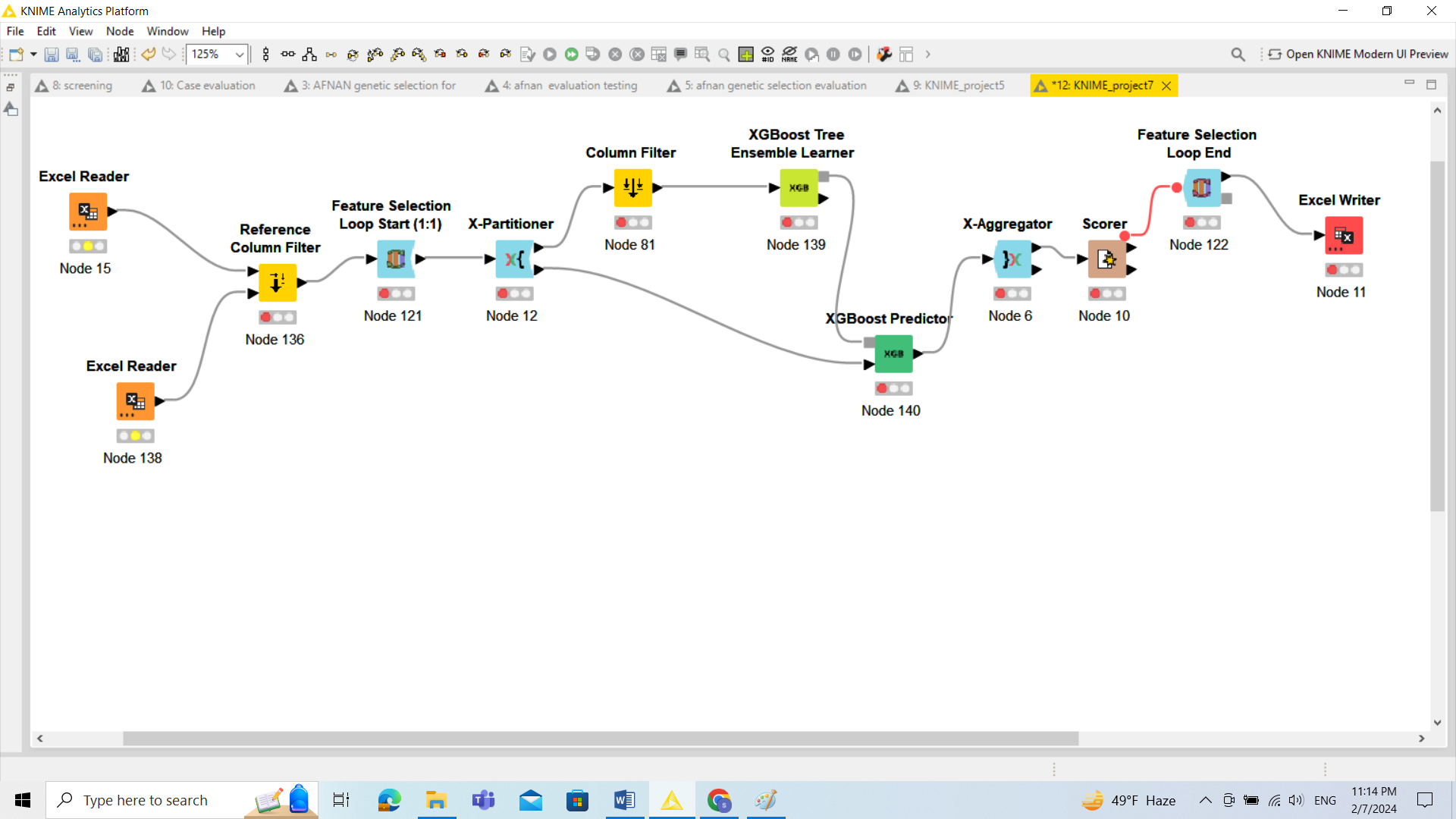


**Figure S6.** Genetic function workflow using XGBoost.


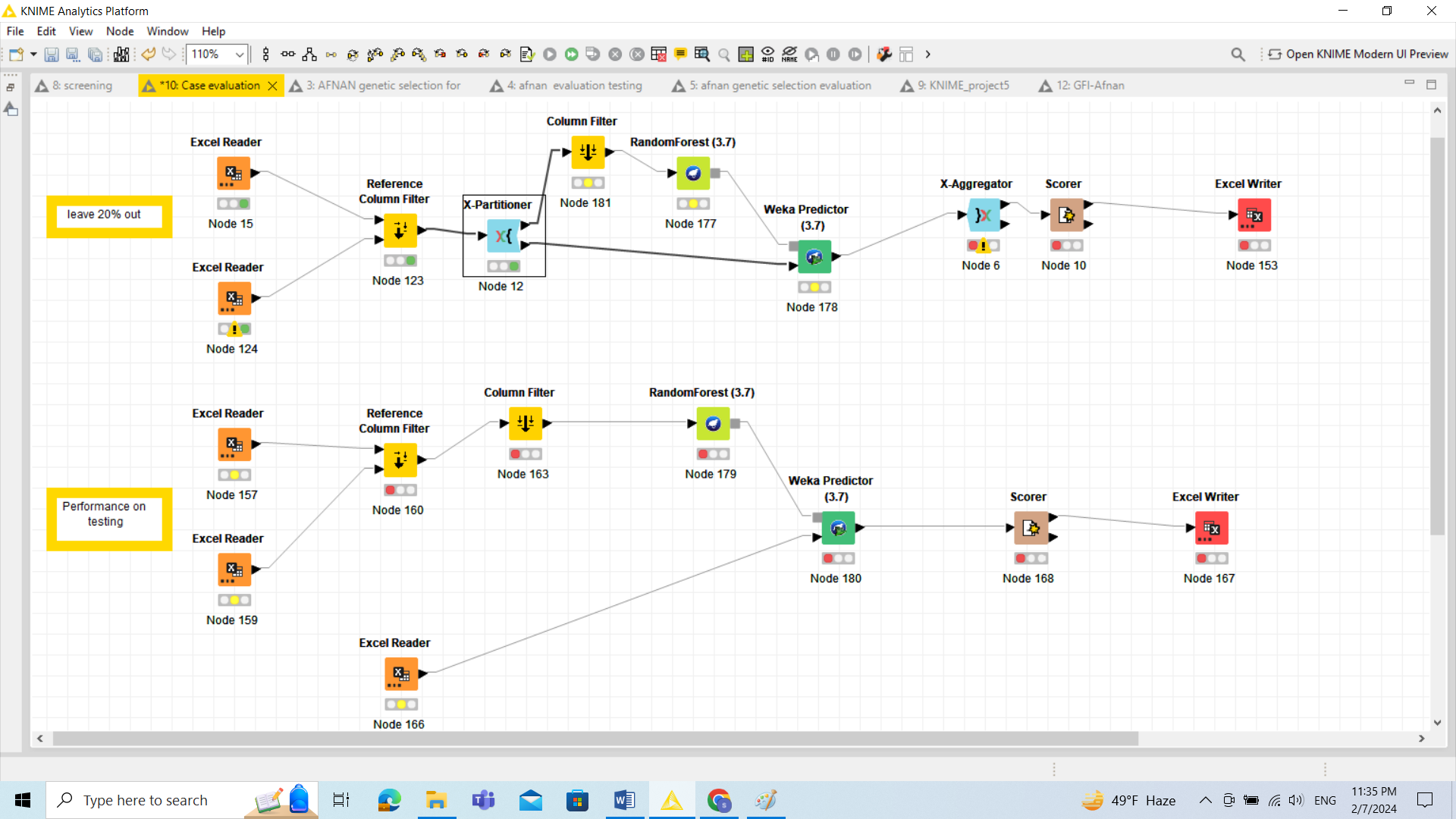


**Figure S7.** Evaluation the successful random forest model against training and testing lists.


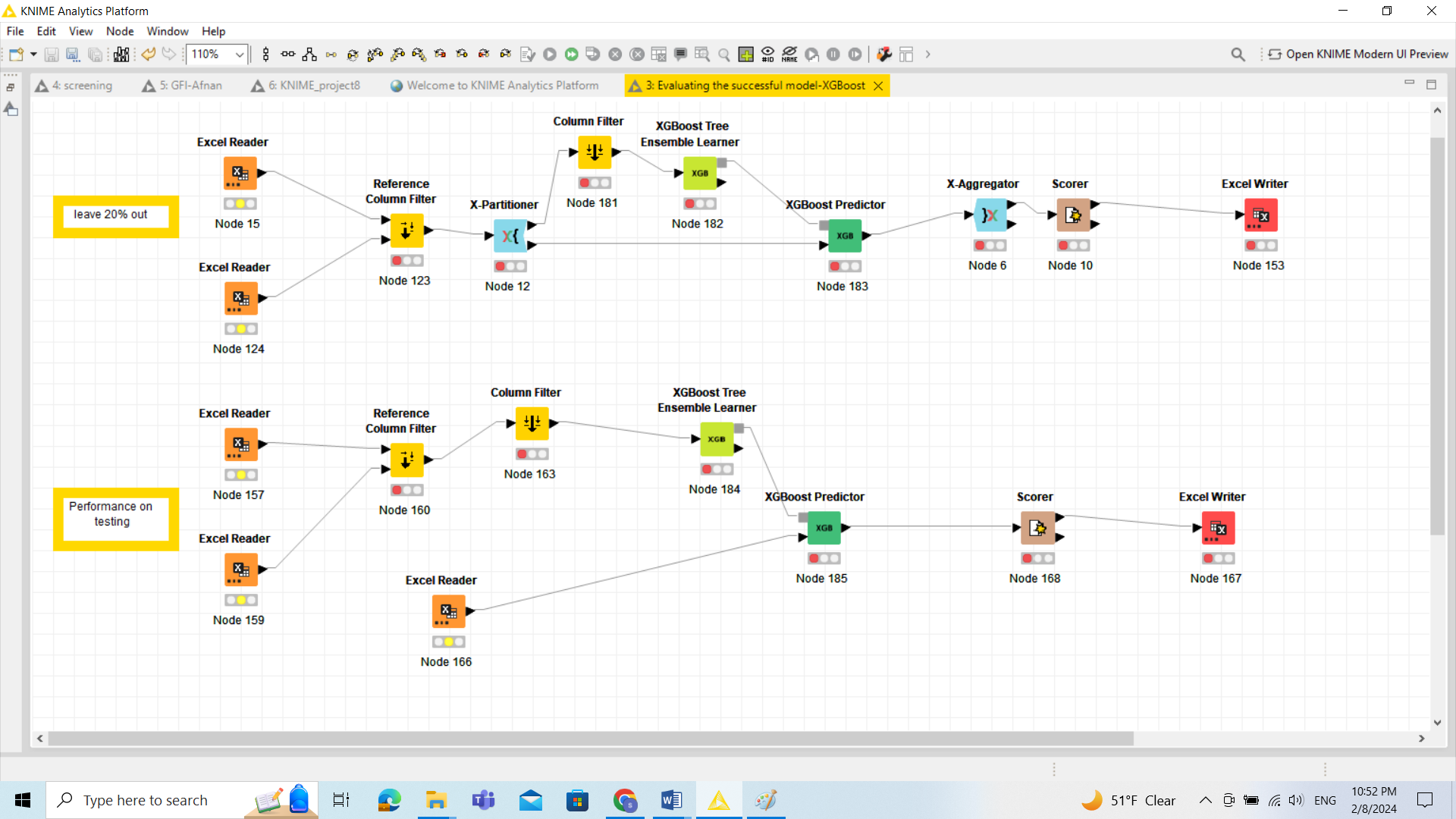


**Figure S8.** Evaluation the successful XGBoost model against training and testing lists.


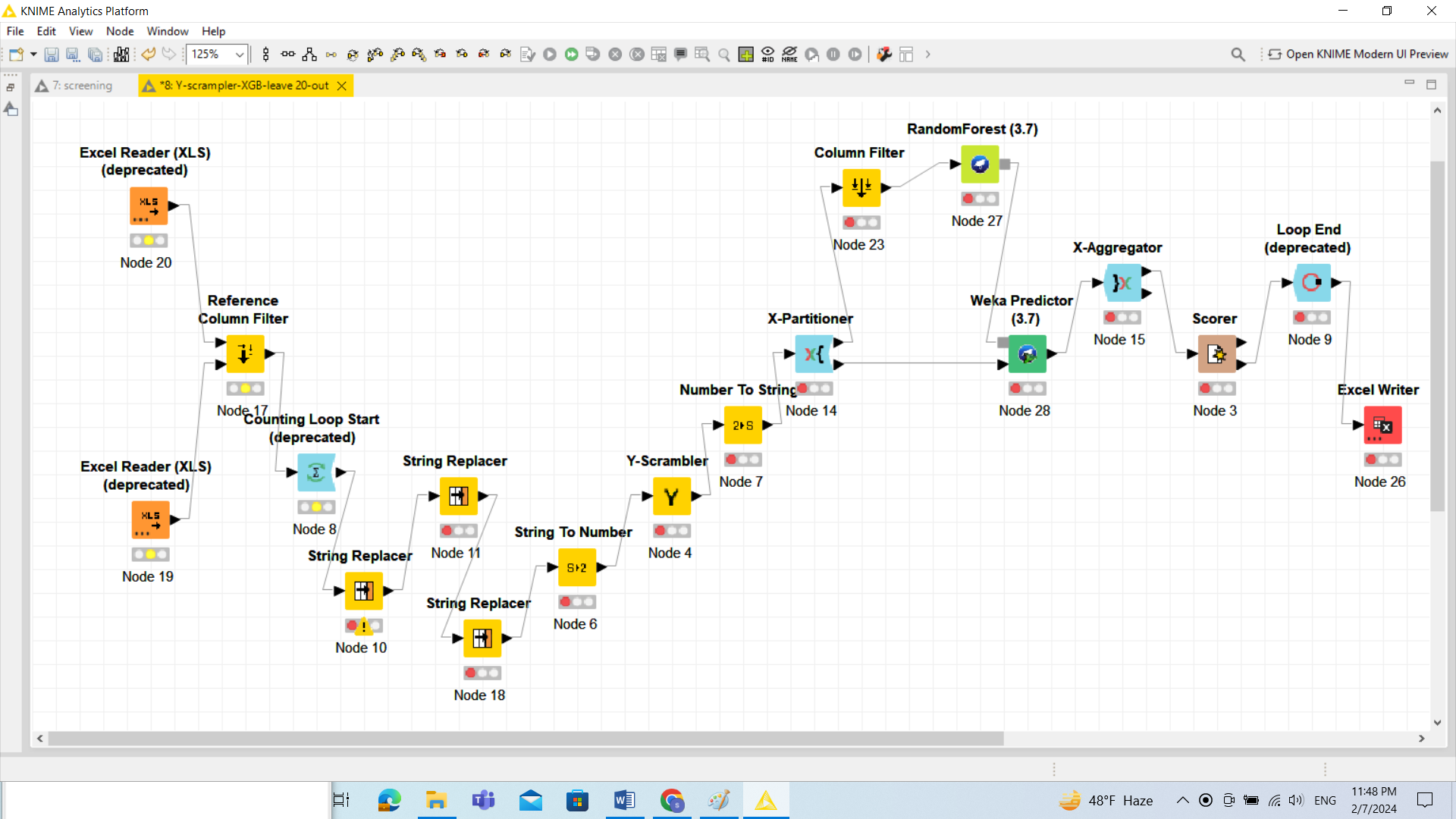


**Figure S9. Y-scrambling workflow for the successful random forest model.**


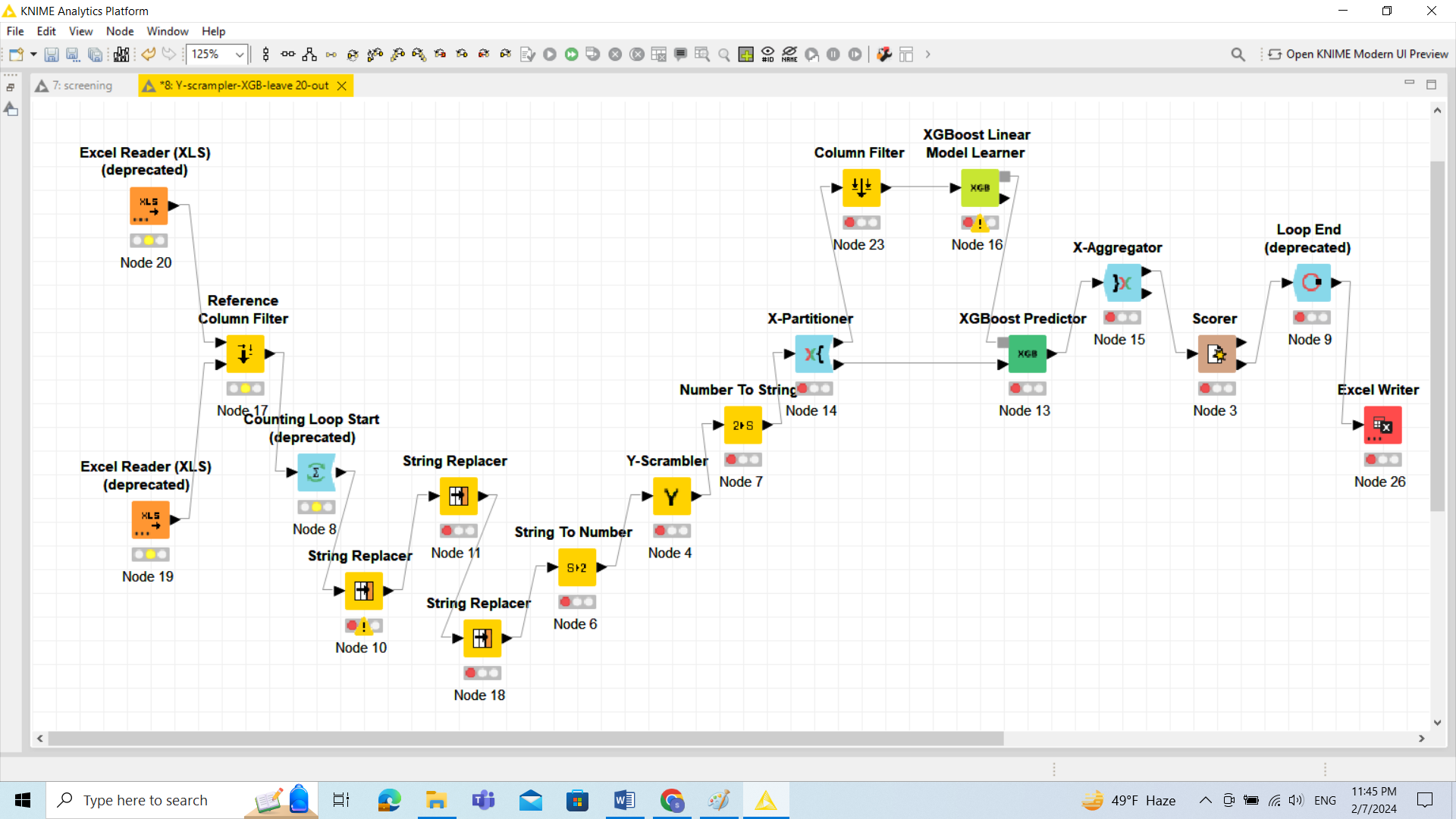


**Figure S10. Y-scrambling workflow for the successful XGBoost model.**


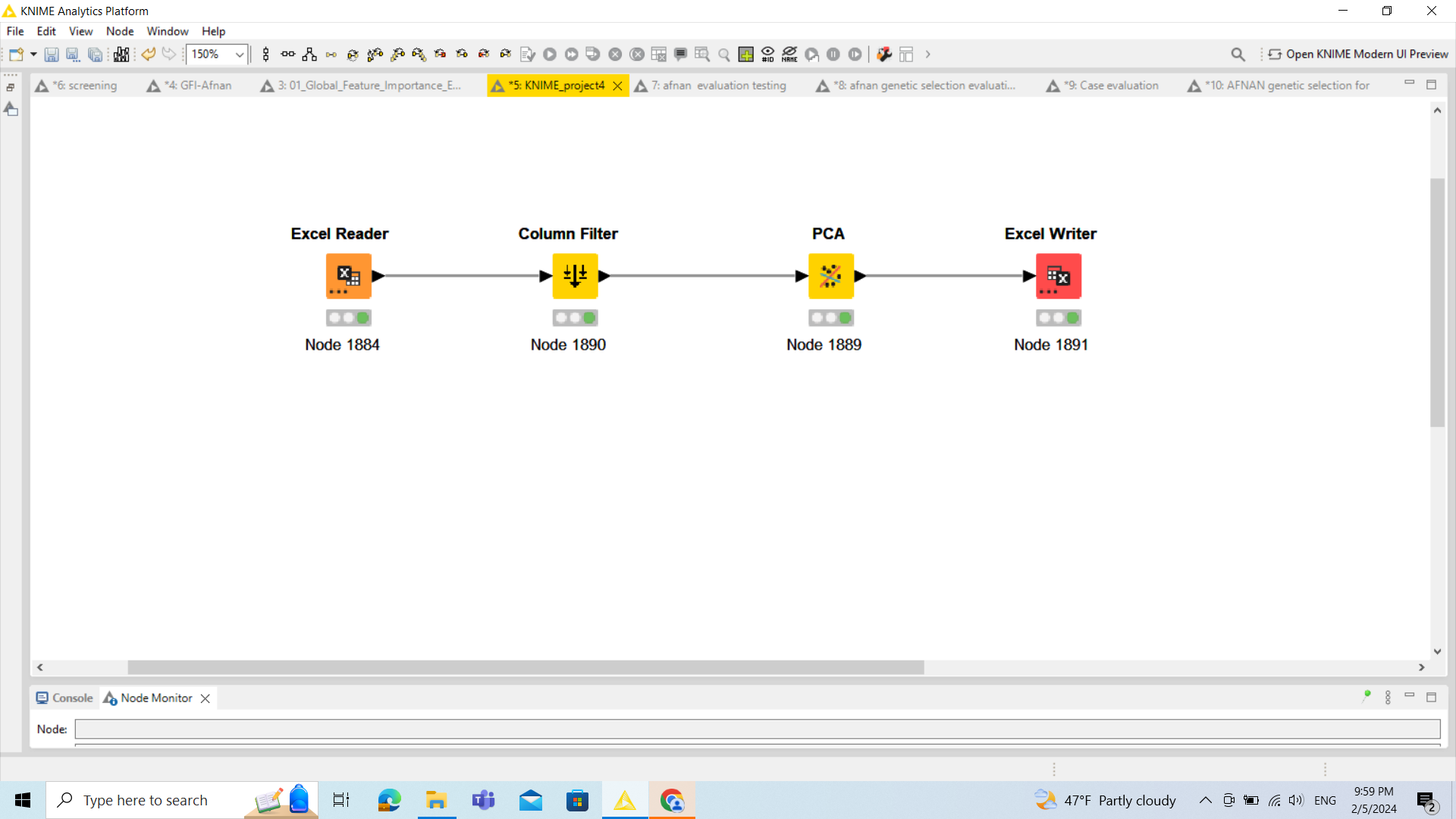


**Figure S11.** Principle component analysis workflow implemented in this study.
